# Automated Synthesis of a 96 Product-Sized Library of Triazole Derivatives Using a Solid Phase Supported Copper Catalyst

**DOI:** 10.3390/molecules15053087

**Published:** 2010-04-28

**Authors:** Ibtissem Jlalia, Claire Beauvineau, Sophie Beauvière, Esra Önen, Marie Aufort, Aymeric Beauvineau, Eihab Khaba, Jean Herscovici, Faouzi Meganem, Christian Girard

**Affiliations:** 1 Laboratoire de Pharmacologie Chimique et Génétique (UMR 8151 CNRS, U 1022 INSERM, IFR 2769), Ecole Nationale Supérieure de Chimie de Paris – Chimie ParisTech; 11, rue Pierre et Marie Curie, 75005 Paris, France; E-Mail: ibtissemj@yahoo.fr (I.J.); 2 Laboratoire de Synthèse Organique et Application, Faculté des Sciences de Bizerte, Université du 7 Novembre à Carthage, 7021 Jarzouna, Bizerte, Tunisia; E-Mail: Faouzi.Meganem@fsb.rnu.tn (F.M.)

**Keywords:** click chemistry, Huisgen cycloaddition, copper (I) catalysis, triazoles, supported catalyst, parallel synthesis

## Abstract

This article deal with the parallel synthesis of a 96 product-sized library using a polymer-based copper catalyst that we developed which can be easily separated from the products by simple filtration. This gave us the opportunity to use this catalyst in an automated chemical synthesis station (Chemspeed ASW-2000). Studies and results about the preparation of the catalyst, its use in different solvent systems, its recycling capabilities and its scope and limitations in the synthesis of this library will be addressed. The synthesis of the triazole library and the very good results obtained will finally be discussed.

## 1. Introduction

With creativity, the organic chemist is seeking new structures and methods to access molecular diversity. Over the years of organic synthesis adventure, several reagents and routes have been discovered. They were applied in total synthesis of natural compounds, or analogs, and to elaborate entirely new structures. Many efficient tools are now available to the chemist who wants to play with chemical architecture. Nevertheless the effectiveness of all the available approaches, there is still a need for new methods and approaches that can improve both efficiency and speed of preparation of organic derivatives.

**Scheme 1:**
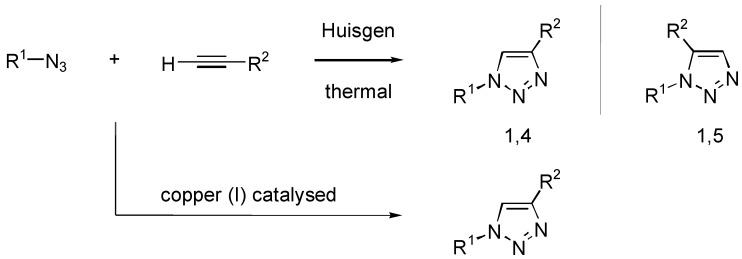
Huisgen cycloaddition route to 1,2,3-triazoles and its copper (I) catalyzed version.

Click-chemistry. The use of fast and efficient reactions to access molecular diversity in (almost) a blink of an eye. This concept also gives the opportunity to play with molecular sub-units to create molecular architecture with infinite creativity. The click-chemistry philosophy is to identify fast, efficient and general reactions using very reactive partners. Such reactions can then be applied towards synthesis of highly diversified structures, using a few reactions, with quasi-infinite possibilities by varying the partners. Chemists in all fields are now using this concept and the reactions that have been identified as "clickable" [1,2,3,4,5]. Among the reactions that have been studied so far, one of the best examples of the click-chemistry concept is the [3+2] cycloaddition between azides an alkynes in its copper (I)-catalyzed version. The classical thermal Huisgen’s cycloaddition reaction [6], to give access to 1,4- and 1,5-disubstituted 1,2,3-triazoles, was greatly improved by the use of cuprous salts [7,8,9,10,11,12,13,14]. Furthermore, this new set of conditions permits the reaction to be conducted at lower temperature, in various solvents and to regioselectively obtain the 1,4-disubstituted isomer (Scheme 1)

Many conditions have been published so far and can be summarized as the addition of copper (I) salts in organic or aqueous solutions often in conjunction with a base [15,16,17], copper (II) salts / ascorbic acid redox system (to generate the copper (I) species *in situ*) [18,19,20], copper (I) salts supported on minerals or polymers [21,22,23,24,25,26,27,28,29], metallic copper alone or adsorbed [30,31,32,33,34,35,36,37] and even copper (II) salts as such or on different supports [38,39,40,41,42].

We published our initial findings on a new and original catalytic system based on copper (I) iodide chelated on Amberlyst A-21 polymer for a use in automated solution synthesis of 1,2,3-triazoles from organic azides and terminal alkynes [43]. The advantages of this catalyst are the ease of preparation, a good catalytic activity and the simple separation from the reaction product by filtration. The first examples seemed to demonstrate that this system had some advantages, making it a good candidate for a use in automated synthesis. We wish to report here our findings and complete studies on this catalyst, as well as its use in the automated parallel synthesis of a 96 compound-sized library in solution.

## 2. Results and Discussion

### 2.1. Preparation of the catalyst

Most of the published methods involving the use of copper salts to catalyze Huisgen’s reaction are conducted in the presence of an added base, which can be of organic or inorganic nature. The presence of a base was suggested to be needed in order to help the formation of an intermediate copper (I) acetylide, which may be formed and accounted for the observed regioselectivity [44,45]. In our initial communication, in order to choose a heterogeneous catalytic system, we decided to look for a polymer which can both chelate copper salts and act as a base. For this purpose we selected Amberlyst A-21, a dimethylaminomethyl-grafted polystyrene, bearing an amine group, which is meeting both criteria [43]. After a first evaluation of the solubility of simple copper (I) halides in various organic solvents, we selected copper (I) iodide in acetonitrile to prepare the catalyst.

**Scheme 2:**
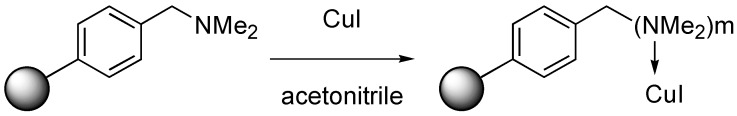
Preparation of the heterogeneous catalyst Amberlyst A-21•CuI.

From the experiments, the better set for the fixation protocol was the use of the ratio 2.4 mmol amine / 1.0 mmol CuI. This gave a light green colored polymer with a final composition of 1.23 mmol CuI/g of resin, indicative of a 2.98 mmol amines for 1 mmol CuI. Elemental analysis of this polymer finally gave a copper content of 8.64%, which gave a loading of 1.36 mmol CuI g^−1^, close to the value measured by the weight increase (Scheme 2) [43].

### 2.2. Influence of the solvent 

**Figure 1:**
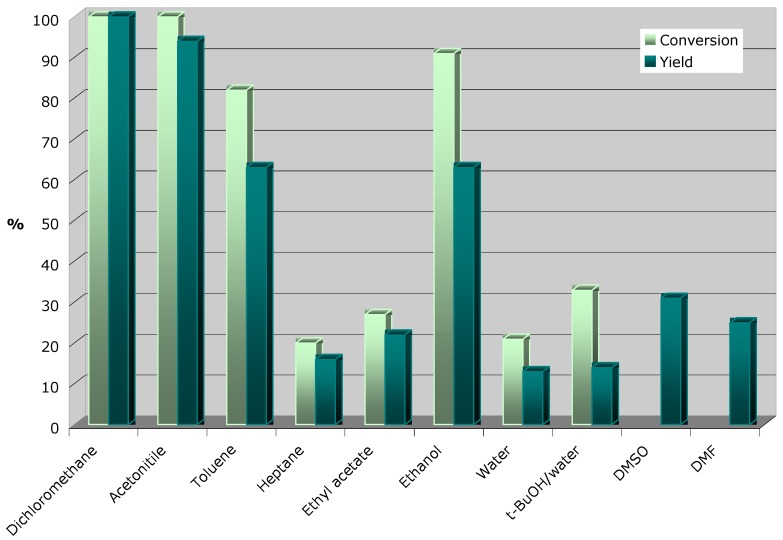
Conversions and yields of triazole **3** in different solvents using 10 mol % A-21•CuI.

In our preliminary study, we found out that the catalyst worked better in methylene chloride than acetonitrile, not always in terms of yields due to triazoles’ solubility but, most of all, this solvent prevented copper from leaching from the catalyst [43]. In order to determine if our catalytic system can be used under other conditions, a study of both conversion and yield, as a function of the solvent used, was done at 10 mol % in catalyst (Figure 1). A panel of solvents as a function of their polarity and nature were selected and the results studied by NMR for conversion (C) and isolated yields (Y) for an over-night on the model reaction (16 h) for the formation of **3**. In case of dichloromethane (C: 100%, Y: 100%), the product was totally formed and isolated pure after a simple filtration. For acetonitrile (C: 100%, Y: 94%), the product needed a purification step in order to remove some copper salts that leached. The reaction seemed to work well in toluene (C: 82%, Y: 63%) but the yield was lower after removal of the excess azide **2**. In heptane (C: 20%, Y: 16%) and ethyl acetate (C: 27%, Y: 22%) both yields and conversions were low. This can be due to poor solubility of the reagents in these solvents.

Interestingly, the reaction proceeds with a good conversion but a modest yield in ethanol (C: 91%, Y: 63%). This can be due to a partial solubility of the reagents in ethanol. In aqueous systems such as water (C: 21%, Y: 13%) and t-BuOH/water (1/1) (C: 33%, Y: 14%) the conversions were low as well as yields after purification.

For the polar and high boiling DMSO (Y: 31%) and DMF (Y: 25%) the yields were only measured after cumbersome extraction and residual solvent evaporation followed by purifications. From this study, it is obvious that all reagents have to be at least a little soluble in the reaction media in order to react at polymer’s surface, where the catalyst is fixed. In very polar and protic solvents, not very suitable for use with poly(styrene) derivatives, the conversion and yields ranged from 10–30%. Furthermore, more difficult isolation and purification procedures were needed and some copper leaching was always observed when the crude samples were analyzed.

### 2.3. Influence of the catalyst amount 

**Figure 2:**
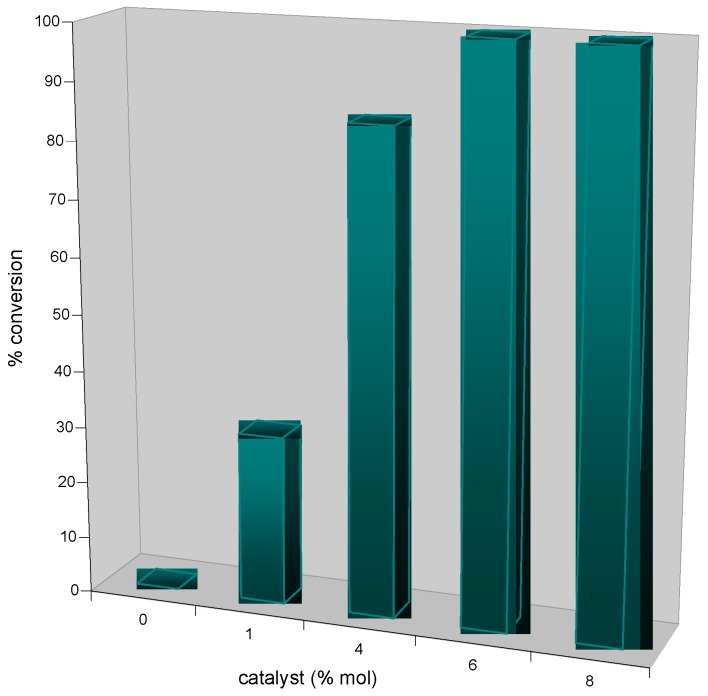
Yield as a function of mol % A-21•CuI.

In our first publication and studies presented here, we selected arbitrarily an amount of 10 mol % to conduct reactions [43]. We then found out that the catalyst was better working in methylene chloride, rather than other solvents, as discussed in the previous section. Thus, a study of conversion as a function of the catalyst ratio was conducted in deuteriochloroform and the results are presented in Figure 2. When the catalyst amount was varied from 1 to 8 mol %, the conversion gradually increased from 30 (1 mol %) to 85 (4 mol %) and finally reached a quantitative value for 6 and 8 mol % for an over-night reaction.

Even if 6 mol % was enough to obtain a quantitative yield of **3**, the value of 8 mol % was selected to insure a complete transformation in most of the reactions using this procedure and reaction time. Of course, an amount of 10 mol %, as we previously used, can guarantee as well a complete transformation of most of the reaction partners.

### 2.4. Reaction kinetics: homogeneous vs. heterogeneous conditions 

**Scheme 3:**
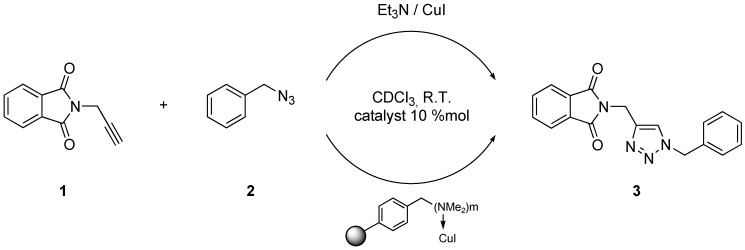
Reaction in homogeneous and heterogeneous conditions for the formation of **3**.

In order to compare the efficiency of our catalytic system, kinetics studies were done on the model reaction with Amberlyst A-21•CuI and the Et_3_N/CuI couple at the same catalytic level, *i.e.* at 10 mol % (Scheme 3). The reactions were followed-up by NMR analysis of the reaction mixtures using deuteriochloroform as the solvent.

**Figure 3:**
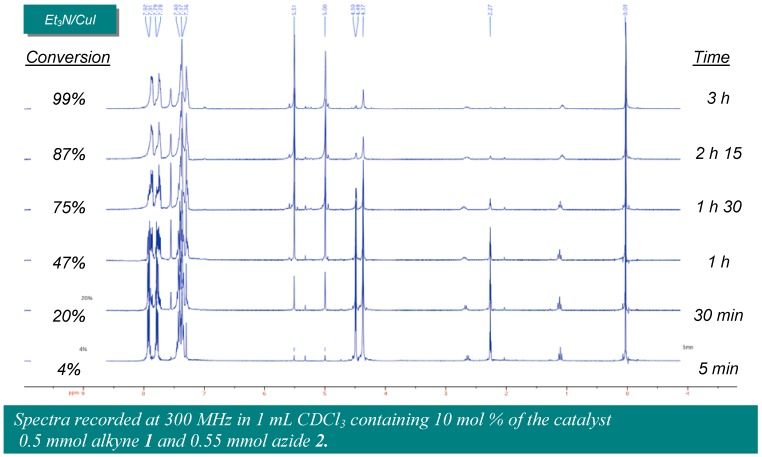
NMR follow-up in homogeneous conditions (Et_3_N/CuI in CDCl_3_) for the formation of **3**.

**Figure 4:**
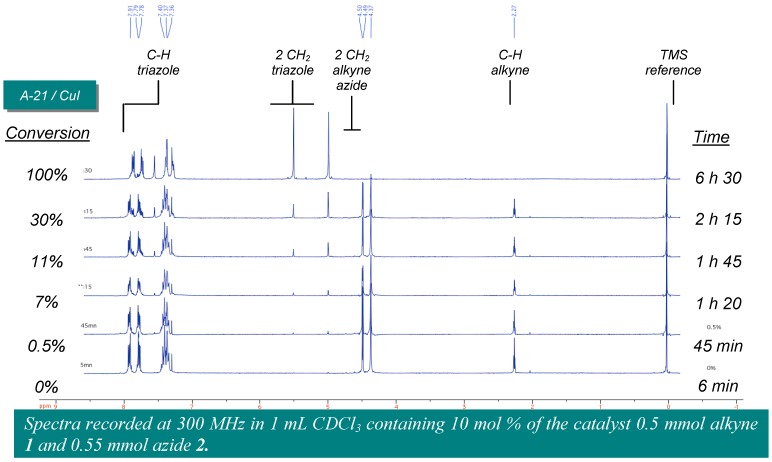
NMR follow-up in heterogeneous conditions (A-21•CuI in CDCl_3_) for the formation of **3**.

The NMR spectra for both experiments are presented in Figure 3 and Figure 4. The reaction is easily followed by the disappearance of the alkyne **1** proton (triplet at 2.27 ppm) that is replaced by the H-5 proton of the formed triazole **3** (singlet, 7.51 ppm). The methylene protons of the starting azide **2** (singlet, 4.37 ppm) and alkyne **1** (doublet, 4.50 ppm) are shifted downfield and appear as singlets at 5.00 and 5.51 ppm respectively.

**Figure 5:**
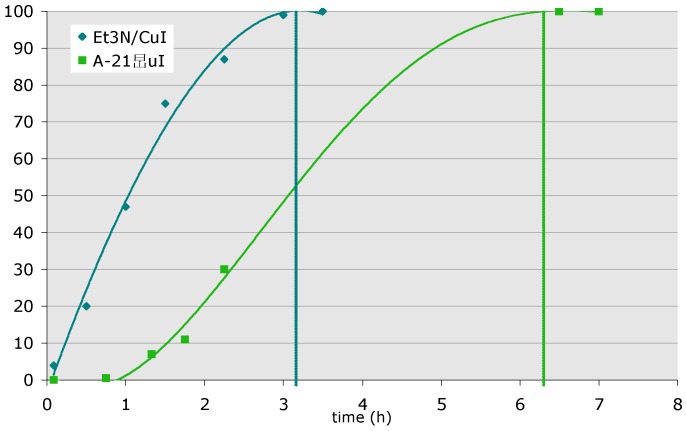
Kinetics for the formation of **3** in homo and heterogeneous conditions.

For the homogeneous conditions (Et_3_N/CuI) the reaction started as soon as the reagents were mixed (Figure 3, Figure 5). After only five minutes, there was already around 5% conversion. The reaction is half completed after one hour and total conversion was reached in a little more than three hours (quantitative for the 3.5 h sample). In the case of the Amberlyst A-21•CuI catalyst, in heterogeneous conditions (Figure 4, Figure 5), there was an induction time of around an hour (0.5% conversion after forty-five minutes). The reaction then proceeded smoothly, reaching half completion after three hours, and was over after six hours and a half.

By comparison, using the model reaction leading to the formation of **3**, the homogeneous conditions were twice as fast as the heterogeneous ones. However, the isolation procedures were different and in favor of the insoluble catalyst. The use of A-21•CuI only necessitated a filtration and evaporation of the solvent, the product **3** being isolated in quantitative yield. In the case of the reaction with Et_3_N/CuI, extractions and purification were needed but the product **3** was isolated in a good yield of 75%.

### 2.5. Recycling and stability of the catalyst 

Since the catalyst is recovered at the end of reaction, its reuse (recycling) and stability were finally evaluated (Table 1). Another model reaction was used for these tests, namely the formation of 1-carboethoxymethyl-4-carbomethoxy-1,2,3-triazole (**6**) from methyl propiolate (**4**) and ethyl azidoacetate (**5**).

Recycling of the catalyst was done in two ways, but for both of them the catalyst was recovered by filtration, washed with the reaction solvent (CH_2_Cl_2_) and dried under vacuum before being reused immediately for another cycle or kept in a vial before to reuse it.

In the first series of experiments (Entries 1 to 6), the catalyst was immediately reused after a first reaction. The catalyst was thus reused over five days (one reaction a day) without losing its efficiency or activity leading to **6** in quantitative yields.

For the second series (Entries 7 to 12), the catalyst was kept on the shelve between the reactions. The reactions were then done each week over a five-week period. Once again, the catalyst leads to the formation of **6** in a quantitative manner with no differences between the cycles.

Interestingly, the stability of this catalyst seem very good, since it can even be used after one year without lost in the activity and selectivity. A possible explanation is that chelation of the copper by the polymer amine “ligands” (-CH_2_NMe_2_) could be accounted for this stability, maybe by preventing oxidation of the copper (I) state [12].

### 2.6. Application: Automated synthesis of a library of 96 triazoles 

**Scheme 4:**
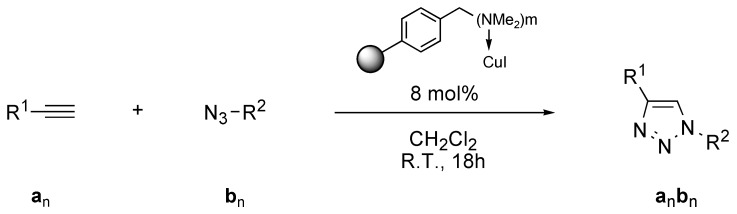
General scheme for automated triazoles **a_n_b_n_** synthesis from alkynes **a_n_** and azides **b_n_**.

In order to evaluate scope and limitations of this catalytic system, the automated synthesis of a library of 96 triazoles was undertaken (Scheme 4). The synthetic operations were conducted using a ChemSpeed ASW-2000 machine by loading the catalyst (8 mol%) in the reactor and injecting dichloromethane solutions of twelve alkynes **a_n_** and eight azides **b_n_**. After 18 h of orbital agitation at room temperature, the reaction mixtures were filtered and the corresponding ninety-six triazoles **a_n_b_n_** recuperated in solution. The results are presented in Table 2.

When looking at the alkynes **a_n_** used, propargyl alcohol (**a1**) and *N*-acetylpropargylamine (**a6**) gave equal average yields of 74%; methyl propiolate (**a4**), phenylacetylene (**a10**), 1-decyne (**a11**) and trimethylsilylacetylene (**a12**) average yields between 80–85%; and finally phenyl propargyl ether (**a2**), propiolaldehyde diethyl acetal (**a3**), *N*-propargylphthalimide (**a5**), *N*-trifluoroacetylpropargylamine (**a7**) and *N*-(*t*-butoxycarbonyl)propargylamine (**a8**) average yields within 90–95% range. Tripropargylamine (**a9**) was the only alkyne that did not give good results. The problem is arising from the fact that the three alkyne functions have to react to form the corresponding tris(triazolylmethyl) amines. These conditions did not permit the complete formation of the tris compounds giving various ratios of tris (T), di (D) and mono (M) triazole derivatives. Furthermore, the presence of the amine center in the molecules was found to cause copper leaching at some extent. This outcome is quite different from the results obtained without solvent with this catalyst, on in solution with a clay supported one, where the tris(triazolylmethyl) amines were exclusively obtained [23,46].

If the results are analyzed by looking at the partner azides **b_n_** in the reaction; 3-azidopropanol (**b3**), 3-azido-*N*-trifluoroacetylpropylamine (**b4**) and 2-(2-azidoethyl)thiophene (**b7**) gave average yields of 80%; 1-azido-1-deoxy-2,3-*O*-isopropylideneglycerol (**b5**) and 3,6-anhydro-1-azido-1-deoxy-4,5-*O*-isopropylidene-D-glucitol (**b8**) near 85%; and finally for benzyl azide (**b1**), ethyl azidoacetate (**b2**) and cyclohex-3-enemethyl azide (**b6**) the yields were around 90%.

Most of the reactions for this library generation gave good yield of triazoles, with an average of 85%. For the 96 products isolated, excluding tripropargylamine (**a9**), 74% of the library gave yields between 80–100%, 18% of it yields between 60–80%, and only 4% and 3% of the yields were between 50–60% and below 50% respectively. The lowest yields observed here are usually due to a poor solubility of the triazole in dichloromethane, especially for oily products that tend to coat the polymer beads. We indicated in Table 2 yields obtained after filtration and washings of the polymer by dichloromethane. The yields can be increased in the case of lower solubility by doing acetonitrile washes, but with the cost of variable levels of pollution of the triazole by copper leaching from the catalyst.

In all reactions, only the 1,4-isomer was observed and all products save some exceptions were pure. As previously observed with this system, no azide excess was found into the reaction products obtained in quantitative yields, suggesting a possible sequestration by the polymer. No copper leaching was observed in most cases. The products were therefore isolated as pure after a simple filtration/ evaporation procedure. For reactions were the excess azide was still present or some copper leaching occurred, the impurities can be easily removed from products’ solutions in dichloromethane using polymer-supported triphenylphosphine and thiourea [47,48,49,50,51].

## 3. Experimental 

### 3.1. General methods

*Chemicals:* Copper (I) idodide, propiolaldehyde diethyl acetal (**a3**) and propiolic acid methyl ester (**a4**) were purchased from Lancaster; propargyl alcohol (**a1**), tripropargylamine (**a9**), 1-decyne (**a11**) and trimethylsilylacetylene (**a12**) from Aldrich; phenylacetylene (**a10**) form Alfa-Aesar and used without further purification. Phenyl propargyl ether (**a2**) and propargyl phthalimide (**a5**) were prepared from propargyl bromide and phenol and potassium phthalimide respectively [55,56,57]. *N*-protected derivatives of propargylamine (acetyl (**a6**), trifluoroacetyl (**a7**) and *t*-butoxycarbonyl (**a8**)) were synthesized from the amine and the corresponding anhydrides following standard procedures.

Azides **b1-4** were prepared from sodium azide and benzyl bromide, ethyl bromoacetate, 3-chloropropanol and 3-bromopropylamine hydrobromide (after treatment with ethyl trifluoroacetate) following published procedures [58,59,60,61]. Azides **b5-7** were obtained from a standard sequential mesylation and azide displacement procedure starting from the corresponding commercially available alcohols. Azide **b8** was obtained by iodide substitution by azide on the corresponding iodo derivative, easily accessible in one step from isosorbide [62].

*Solvents:* Acetonitrile (spectrometric grade, low water), toluene, heptane, ethyl acetate, *t*-butanol and ethanol was purchased from SDS France and used as such. Dimethylsulfoxide and dimethylformamide (spectrometric grade, low water) were placed oven 4 Å molecular sieves and kept under nitrogen. Dichloromethane (SDS France) was treated with phosphorus pentoxide at reflux (1 h) before being distilled.

*Melting points (m.p.):* Melting points were determined using a Kofler apparatus after a first evaluation, calibration with a reference sample of a mp near the observed fusion and final measure of the melting point.

*Infrared spectroscopy (IR):* Infrared spectra were recorded neat on a Jasco FT/IR-4100 in ATR mode (PIKE-MIRacle) between 4 000 and 400 cm^−1^ and are given in *ν* (cm^−1^)

*Nuclear Magnetic Resonance (NMR):* NMR spectra were recorded on a Bruker Avance DRX at 300 MHz for ^1^H and 75.5 for ^13^C. Chemical shifts (δ) are reported in part per million (ppm) relative to tetramethylsilane signal as an internal reference. Couplings constants (*J*) are in hertz and signal multiplicities indicated as s (singulet), d (doublet), t (triplet), q (quadruplet), m (multiplet), dd (doublet of doublet).

*Liquid Chromatography / Mass Spectrometry (LC-MS):* LC-MS analyses were done on a Shimadzu LCSM-2010 A on a HPLC, column Alltima HP C8 3μ (Alltech), reversed phase (L = 53 mm; ID = 7 mm), PDA diodes detector SPD-M10 A (D_2_, lamp from 190 to 400 nm) and light scattering detector ELSD-LT. The LC were run using a 1 ml/min flow using a gradient between acetonitrile and water containing formic acid (0,1%): 0 to 1 min: 30% CH_3_CN, 1 to 5 min: from 30% to 100% CH_3_CN, 5 to 12 min: 100% CH_3_CN, 12 to 14.99 min: from 100% to 30% CH_3_CN, 14.99 to 20 min: 30% CH_3_CN. MS spectrum was recorded between *m/z* = 100 to 500 at the exit of the column using an ESI ionization and positive ion mode (detector= 1.5 kV, quadripole = 5 V).

### 3.2. Procedures

*Dry Amberlyst A-21* [43]: Commercial wet Amberlyst A-21 resin (Aldrich, 20–50 mesh, 100 g) was suspended in MeOH (500 mL) for 0.5 h and filtered (3 times) and then soaked in methylene chloride (500 mL) for 0.5 h and again filtered (3 times). The resulting resin was placed in a round-bottom flask on a rotatory evaporator and dried at 50 ºC under 10 mm Hg until it was free flowing. The dried resin was then kept overnight *in vacuo* in a desiccator over P_2_O_5_. Specifications from the manufacturer indicate that the polymer contains 4.8 mequiv of amine/g of dry resin.

*Preparation of the supported catalyst (A-21.CuI)* [43]: Dry Amberlyst A-21 (1.0 g, 4.8 mmol amine) was added to a solution of copper (I) iodide (381 mg, 2.00 mmol) in acetonitrile (15 mL) and gently shaken on an orbital stirrer for 17 h. The solvent was drawn off and the resin washed with CH_3_CN (2 × 15 mL), CH_2_Cl_2_ (2 × 15 mL) and dried in vacuo (0.01 mm Hg) at 40 ºC. The weight increase was of 0,307 g (1.61 mmol CuI) that gave a polymer loading of 1.23 mmol CuI.g^−1^. Elemental analyses (Service Central d’Analyses du CNRS, Solaize, France) gave a copper content of 8.64%, indicative of a loading of 1.35 mmol CuI.g^−1^.

General procedure for automated synthesis of triazoles

Amberlyst A-21·CuI (1.35 mmol/g, 30 mg, 0.040 mmol, 8 mol %) was placed in one of the Chemspeed ASW-2000 reactor equipped with a plunging filter, leaving the paired reactor empty. The azide (0.55 mmol) and alkyne (0.50 mmol), both dissolved in 1 mL dichloromethane were sequentially added at 1mL·min^−1^. The reactors were orbitally shaken at 600 rpm for 12 hours at room temperature. The product’s solution was separated from the catalyst by filtration followed by washing of the catalyst by dichloromethane (2 × 2.5 mL). The combined extracts were evaporated to obtain the product.

**CAUTION!** Organic azides are potentially explosive and should be handle with care. Even if no incident occurred in this reaction on this scale, the cycloaddition can be exothermic and should not be attempted on a larger scale, without being aware of explosion risks.

*1-Benzyl-4-hydroxymethyl-1,2,3-triazole* (**a1b1**). Prepared from 28 mg (0.50 mmol) of **a1** and 73 mg (0.55 mmol) of **b1**. The product was obtained as a white solid (92 mg, 97%). C_10_H_11_N_3_O, M = 189.22 g.mol^−1^. m.p. 76–78 ºC. FTIR: *ν* 3257, 3144, 3091, 1451, 1040 cm^−1^. ^1^H-NMR (CDCl_3_): *δ* 4.47 (s, 1H), 4.70 (s, 2H), 5.46 (s, 2H), 7.17–7.45 (m, 5 H), 7.91 (s, 1H) ppm. ^13^C-NMR (CDCl_3_): *δ* 54.1, 56.0, 122.0, 128.1, 128.7, 129.1, 134.5, 148.0 ppm. LC-MS: ELSD pur. 97%, UV pur. 100%, R_t_ = 3.41 min, *m/z*: 190 ([M+H]^+^, 100%).

*1-Ethoxycarbonylmethyl-4-hydroxymethyl-1,2,3-triazole* (**a1b2**). Prepared from 28 mg (0.50 mmol) of **a1** and 71 mg (0.55 mmol) of **b2**. The product was obtained as a pale yellow oil (92 mg, 99%). C_7_H_11_N_3_O_3_, M = 185.18 g.mol^−1^. FTIR: *ν* 3110, 3076, 3038, 2849, 1708 cm^−1^. ^1^H-NMR (CDCl_3_): *δ* 1.26 (t, *J* = 7.2 Hz, 3H), 4.21 (q, *J* = 7.2 Hz, 2H), 4.72 (s, 2H), 5.12 (s, 2H), 7.67 (s, 1H) ppm. ^13^C-NMR (CDCl_3_): *δ* 14.0, 50.8, 56.1, 62.4, 123.8, 148.3, 166.5 ppm. LC-MS: ELSD pur. 99%, UV pur. 100%, R_t_ = 3.02 min, *m/z*: 186 ([M+H]^+^, 81%).

*4-Hydroxymethyl-1-(3-hydroxypropyl)-1,2,3-triazole* (**a1b3**). Prepared from 28 mg (0.50 mmol) of **a1** and 56 mg (0.55 mmol) of **b3**. The product was obtained as a viscous colorless oil (37.7 mg, 48%). C_6_H_11_N_3_O_2_, M = 157.17 g.mol^−1^. FTIR: *ν* 3382, 3142, 2944, 2881, 1658, 1437, 1344, 1219, 1138, 1056 cm^−1^. ^1^H-NMR (CD_3_)_2_CO): *δ* 2.02 (q, *J* = 6.0 Hz, 2H), 3.24 (s, 1H), 3.49 (t, *J* = 6.0 Hz, 2H), 4.41 (t, *J* = 6.0 Hz, 2H), 4.63 (s, 2H), 7.66 (s, 1H) ppm. ^13^C-NMR (CD_3_)_2_CO): *δ* 33.8, 47.5, 56.5, 58.8, 122.8, 148.9 ppm. LC-MS: ELSD pur. 90%, UV pur. 100%, R_t_ = 2.67 min, *m/z*: 158 ([M+H]^+^, 100%).

*1-(3-Trifluoroacetamidopropyl)-4-hydroxymethyl-1,2,3-triazole* (**a1b4**). Prepared from 28 mg (0.50 mmol) of **a1** and 108 mg (0.55 mmol) of **b4**. The product was obtained as a grey solid (53 mg, 42%). C_8_H_11_F_3_N_4_O_2_, M = 252.20 g.mol^−1^. m.p. 91 ºC. FTIR: *ν* 3306, 3219, 3061, 2946, 1721, 1576, 1467, 1181, 1069 cm^−1^. ^1^H-NMR (CD_3_)_2_SO): *δ* 2.04 (m, 2H), 3.20 (q, *J* = 6.0 Hz, 2H), 4.37 (t, *J* = 6.9 Hz, 2H), 4.50 (d, *J* = 5.4 Hz, 2H), 5.14 (t, *J* = 5.7 Hz, 1H), 7,98 (s, 1H), 8.49 (s, 1H) ppm. ^13^C-NMR (CD_3_)_2_SO): *δ* 28.9, 36.6, 38.6, 46.8, 55.0, 122.7 ppm. LC-MS: ELSD pur. 99%, UV pur. 100%, R_t_ = 2.07 min, *m/z*: 253 ([M+H]^+^, 100%).

*4-Hydroxymethyl-1-(2,2-dimethyl-1,3-dioxolan-4-yl)methyl-1,2,3-triazole* (**a1b5**). Prepared from 28 mg (0.50 mmol) of **a1** and 86 mg (0.55 mmol) of **b5**. The product was obtained as a yellow oil (89 mg, 90%). C_9_H_15_N_3_O_3_, M = 213.24 g.mol^−1^. FTIR: *ν* 3379, 3146, 2986, 2936, 2881, 1647, 1452, 1379, 1223, 1149, 1060 cm^−1^. ^1^H-NMR (CDCl_3_): *δ* 1.32 (s, 3H), 1.37 (s, 3H), 3.73 (dd, *J* = 5.3, 8.7 Hz, 1H), 4.10 (dd, *J* = 6.1, 8.7 Hz, 1H), 4.36–4.56 (m, 3H), 4.74 (s, 2H), 7.73 (s, 1H) ppm. ^13^C-NMR (CDCl_3_); *δ* 25.2, 26.7, 52.5, 56.6, 66.5, 74.1, 110.3, 123.0, 147.6 ppm. LC-MS: ELSD pur. 95%, UV pur. 100%, R_t_ = 2.18 min, *m/z*: 214 ([M+H]^+^, 100%).

*1-[(Cyclohex-3-en-1-yl)methyl]-4-hydroxymethyl-1,2,3-triazole* (**a1b6**). Prepared from 28 mg (0.50 mmol) of **a1** and 75 mg (0.55 mmol) of **b6**.The product was obtained as a brown oil (96,7 mg, 82%). C_10_H_15_N_3_O, M = 193.35 g.mol^−1^. FTIR: *ν* 3286, 3124, 3070, 3029, 2929, 2838, 1654, 1040, 1015 cm^−1^. ^1^H-NMR (CDCl_3_): *δ* 1.32 (m, 2H), 1.75 (m, 2H), 2.02 (m, 2H), 2.20 (m, 1H), 4.27 (d, *J* = 7.3 Hz, 2H), 4.78 (s, 2H), 5.60–5.72 (m, 2H), 7.58 (s, 1H) ppm. ^13^C-NMR (CDCl_3_): *δ* 24.2, 25.2, 28.9, 29.7, 34.8, 52.8, 55.5, 56.2, 122.5, 127.1, 147.9 ppm. LC-MS: ELSD pur. 80%, UV pur. 100%, R_t_ = 3.06 min, *m/z*: 194 ([M+H]^+^, 100%).

*4-Hydroxymethyl-1-[2-(2-thienyl)ethyl]-1,2,3-triazole* (**a1b7**). Prepared from 28 mg (0.50 mmol) of **a1** and 84 mg (0.55 mmol) of **b7**. The product was obtained as a yellow oil (61 mg, 58%). C_9_H_11_N_3_OS, M = 209.27 g.mol^−1^. FTIR: *ν* 3341, 3147, 2928, 2862, 1548, 1433, 1214, 1045, 1008 cm^−1^. ^1^H-NMR (CDCl_3_): *δ* 3.42 (t, *J* = 7.1 Hz, 2H), 4.59 (t, *J* = 7.1 Hz, 2H), 4.73 (s, 2H), 6.74 (d, *J* = 3.3 Hz, 1H), 6.91 (dd, *J* = 3.3 and 5.0 Hz, 1H), 7.16 (d, *J* = 5.1 Hz, 1H), 7.47 (s, 1H) ppm. ^13^C-NMR (CDCl_3_) ; *δ* 30.8, 51.7, 56.1, 122.3, 124.7, 126.2, 127.3, 138.8, 147.8 ppm. LC-MS: ELSD pur. 95%, UV pur. 100%, R_t_ = 2.74 min, *m/z*: 210 ([M+H]^+^, 100%).

*1-(3,6-Anhydro-1-deoxy-4,5-O-isopropylidene-D-glucitol-1-yl)-4-hydroxymethyl-1,2,3-triazole* (**a1b8**). Prepared from 28 mg (0.50 mmol) of **a1** and 126 mg (0.55 mmol) of **b8**. The product was obtained as a yellow oil (115.5 mg, 81%). C_12_H_19_N_3_O_5_, M = 285.30 g.mol^−1^. FTIR: *ν* 3432, 2986, 2928, 2870, 1379, 1268, 1214, 1103 cm^−1^. ^1^H-NMR (CDCl_3_): *δ* 1.31 (s, 3H), 1.47 (s, 3H), 3.19 (dd, *J* = 3.4 and 6.3 Hz, 1H), 3.43 (dd, *J* = 3.0, 5.4 Hz, 1H), 3.45–3.53 (m, 3H), 4.09 (dd, *J* = 3.9, 10.7 Hz, 1H), 4.57 (dd, *J* = 3,0 and 14,2 Hz, 1H), 4.67–4.73 (m, 1H), 4.78–4.86 (m, 2H), 7.78 (s, 1H) ppm. ^13^C-NMR (CDCl_3_): *δ* 24.42, 25.93, 53.19, 69.63, 72.28, 80.77, 81.42, 81.94, 88.11, 112.68 ppm. LC-MS: ELSD pur. 95%, UV pur. 100%, R_t_ = 2.74 min, *m/z*: 210 ([M+H]^+^, 100%).

*1-Benzyl-4-phenoxymethyl-1,2,3-triazole* (**a2b1**). Prepared from 66 mg (0.50 mmol) of **a2** and 73 mg (0.55 mmol) of **b1**. The product was obtained as a white solid (136 mg, 99%). C_16_H_15_N_3_O, M = 265.31 g.mol^−1^. m.p. 125 ºC. FTIR: *ν* 3132, 3016, 2970, 2920, 2866, 1588, 1488, 1239, 1222, 1052 cm^−1^. ^1^H-NMR (CDCl_3_): *δ* 5.18 (s, 2H), 5.51 (s, 2H), 6.90–7.39 (m,10H), 7.52 (s, 1H) ppm. ^13^C-NMR (CDCl_3_): *δ* 54.2, 62.0, 114.8, 121.3, 128.1, 128.8, 129.1, 129.5, 134.5, 144.6, 158.2 ppm. LC-MS: ELSD pur. 100%, UV pur. 100%; R_t_ = 9.10 min; *m/z*: 266 ([M+H]^+^, 100%).

*1-Ethoxycarbonylmethyl-4-phenoxymethyl-1,2,3-triazole* (**a2b2**). Prepared from 66 mg (0.50 mmol) of **a2** and 71 mg (0.55 mmol) of **b2**. The product was obtained as a beige solid (104 mg, 80%). C_13_H_15_N_3_O_3_, M = 261.28 g.mol^−1^. m.p. 116–118 ºC. FTIR: *ν* 3153, 2944, 2962, 2879, 1746, 1596, 1483, 1471, 1401, 1235, 1210, 1177, 1031 cm^−1^. ^1^H-NMR (CDCl_3_): *δ* 1.25 (t, *J* = 7.1 Hz, 3H), 4.23 (q, *J* = 6.8 Hz, 2H), 5.11 (s, 2H), 5.18 (s, 2H), 6.92–7.29 (m, 5H), 7.73 (s, 1H) ppm. ^13^C-NMR (CDCl_3_): *δ* 14.0, 50.8, 61.8, 62.4, 114.8, 121.2, 124.3, 129.5, 144.5, 158.2, 166.2 ppm. LC-MS: ELSD pur. 100%, UV pur. 100%; R_t_ = 8.30 min; *m/z*: 262 ([M+H]^+^, 100%).

*1-(3-Hydroxypropyl)-4-phenoxymethyl-1,2,3-triazole* (**a2b3**): Prepared from 66 mg (0.50 mmol) of **a2** and 56 mg (0.55 mmol) of **b3**. The product was obtained as a yellow oil (120 mg, 99%). C_12_H_15_N_3_O_2_, M = 233.27 g.mol^−1^. FTIR: *ν* 3304, 3132, 3107, 2945, 2870, 1600, 1488, 1239, 1218, 1052 cm^−1^. ^1^H-NMR (CDCl_3_): *δ* 2.10 (q, *J* = 6.1 Hz, 2H), 3.23 (s, 1H), 4.49 (t, *J* = 6.8 Hz, 2H), 5.16 (s, 2H), 6.94–6.97 (m, 3H), 7.24–7.30 (m, 2H), 7.66 (s, 1H) ppm. ^13^C-NMR (CDCl_3_): *δ* 32.6, 47.1, 58.5, 61.9, 114.7, 121.3, 123.2, 129.5, 144.1, 158.2 ppm. LC-MS: ELSD pur. 97%, UV pur. 100%; R_t_ = 3.54 min; *m/z*: 234 ([M+H]^+^, 100%).

*1-(3-Trifluoroacetamidopropyl)-4-phenoxymethyl-1,2,3-triazole* (**a2b4**). Prepared from 66 mg (0.50 mmol) of **a2** and 108 mg (0.55 mmol) of **b4**. The product was obtained as a yellow solid (111 mg, 68%). C_14_H_15_F_3_N_4_O_2_, M = 382.30 g.mol^−1^. m.p. 79 ºC. FTIR: *ν* 3356, 3140, 3102, 2958, 2883, 1704, 1600, 1559, 1488, 1243, 1206, 1168 cm^−1^. ^1^H-NMR (CDCl_3_): *δ* 2.22 (m, 2H), 3.41 (q, *J* = 6.5 Hz, 2H), 4,42 (t, *J* = 6.5 Hz, 2H), 5.17 (s, 2H), 6.95–7.00 (m, 3H), 7.26–7.31 (m, 2H), 7.68 (s, 1H) ppm. ^13^C-NMR (CDCl_3_): *δ* 29.1, 37.0, 47.7, 61.7, 114.7, 117.7, 121.4, 123.3, 129.6, 144.5, 157.6, 158.1 ppm. LC-MS: ELSD pur. 98%, UV pur. 100%; R_t_ = 8.37 min; *m/z*: 329 ([M+H]^+^, 100%).

*1-(2,2-Dimethyl-1,3-dioxolan-4-yl)methyl-4-phenoxymethyl-1,2,3-triazole* (**a2b5**). Prepared from 66 mg (0.50 mmol) of **a2** and 86 mg (0.55 mmol) of **b5**. The product was obtained as a yellow solid (145 mg, 99%). C_14_H_17_N_3_O_3_, M = 289.34 g.mol^−1^. m.p. 99 ºC. FTIR: *ν* 3132, 3082, 2987, 2929, 2870, 1604, 1492, 1380, 1226, 1035 cm^−1^. ^1^H-NMR (CDCl_3_): *δ* 1.34 (s, 6H), 3.73 (dd, *J* = 5.8, 8.9 Hz, 1H), 4.12 (dd, *J* = 6.2, 8.8 Hz, 1H), 5.22 (s, 2H), 6.97–7.00 (m, 3H), 7.26–7.32 (m, 2H), 7.76 (s, 1H) ppm. ^13^C-NMR (CDCl_3_): *δ* 25.2, 26.6, 52.4, 61.9, 66.4, 73.9, 110.2, 114.7, 121.2, 124.0, 129.6, 144.3, 158.2 ppm. LC-MS: ELSD pur. 99%, UV pur. 100%; R_t_ = 8.38 min; *m/z*: 290 ([M+H]^+^, 100%).

*1-[(Cyclohex-3-en-1-yl)methyl]-4-phenoxymethyl-1,2,3-triazole* (**a2b6**). Prepared from 66 mg (0.50 mmol) of **a2** and 75 mg (0.55 mmol) of **b6**. The product was obtained as a brown oil (140 mg, 99%). C_16_H_19_N_3_O, M = 269.35 g.mol^−1^. FTIR: *ν* 3136, 3095, 3028, 2945, 2916, 2841, 1654, 1604, 1501, 1256, 1040 cm^−1^. ^1^H-NMR (CDCl_3_): *δ* 1.25–1.39 (m, 1H), 1.66–1.84 (m, 2H), 1.98–2.28 (m, 4H), 4.29 (d, *J* = 7.3 Hz, 2H), 5.23 (s, 2H), 5.64–5.70 (m, 2H), 6.97-7.00 (m, 3H), 7.27–7.33 (m, 2H), 7.59 (s, 1H) ppm. ^13^C-NMR (CDCl_3_): *δ* 24.1, 25.8, 28.8, 34.7, 55.5, 62.1, 114.8, 121.2, 122.9, 124.8, 127.1, 129.5, 144.3, 158.2. LC-MS: ELSD pur. 90% ; R_t_ = 9.45 min; *m/z*: 270 ([M+H]^+^, 100%).

*4-Phenoxymethyl-1-[2-(2-thienyl)ethyl]-1,2,3-triazole* (**a2b7**): Prepared from 66 mg (0.50 mmol) of **a2** and 84 mg (0.55 mmol) of **b7**. The product was obtained as a yellow solid (142 mg, 99%). C_15_H_15_N_3_OS, M = 285.37 g.mol^−1^. m.p. 98 ºC. FTIR: *ν* 3132, 3086, 2953, 2916, 2879, 1584, 1492, 1243, 1040 cm^−1^. ^1^H-NMR (CDCl_3_): *δ* 3,44 (t, *J* = 7.0 Hz, 2H), 4.61 (t, *J* = 7.0 Hz, 2H), 5.20 (s, 2H), 6.68–6.69 (m, 1H), 6.86–6.89 (m, 1H), 6.95–6.99 (m, 3H), 7.15–7.17 (m, 1H), 7.26–7.32 (m, 2H), 7.40 (s, 1H) ppm. ^13^C-NMR (CDCl_3_): *δ* 30.8, 51.7, 61.9, 114.8, 121.2, 123.0, 124.6, 126.2, 127.2, 129.5, 138.7, 158.1 ppm. LC-MS: ELSD pur. 100%, UV pur. 100%; R_t_ = 9.00 min; *m/z*: 286 ([M+H]^+^, 100%).

1-(3,6-Anhydro-1-deoxy-4,5-O-isopropylidene-D-glucitol-1-yl)-4-phenoxymethyl-1,2,3-triazole 

(**a2b8**): Prepared from 66 mg (0.50 mmol) of **a2** and 126 mg (0.55 mmol) of **b8**. The product was obtained as a yellow oil (172 mg, 95%). C_18_H_23_N_3_O_5_, M = 361.40 g.mol^−1^. FTIR: *ν* 3443, 2986, 2928, 2857, 1601, 1494, 1378, 1210, 1103, 1032 cm^−1^. ^1^H-NMR (CDCl_3_): *δ* 1.36 (s, 3H), 1.53 (s, 3H), 3.13 (dd, *J* = 3.6, 5 Hz, 1H), 3.40–3.54 (m, 3H), 4.07 (dd, *J* = 3.9 Hz, 10.7Hz, 1H), 4.57 (dd, *J* = 3.6 Hz, 14.2Hz, 1H), 4.66–4.73 (m, 1H), 4.77–4.85 (m, 2H), 5.24 (s, 2H), 7.00–7.05 (m, 3H), 7.30–7.37 (m, 2H), 7.87 (s, 1H) ppm. ^13^C-NMR (CDCl_3_): *δ* 24.3, 25.8, 52.5, 61.9, 69.2, 72.6, 80.6, 81.3, 81.5, 112.6, 114.8, 121.2, 124.8, 129.5, 144.0, 158.2 ppm. LC-MS: ELSD pur. 100%, UV pur. 100%; R_t_ = 6.37 min; *m/z*: 362 ([M+H]^+^, 100%).

*1-Benzyl-4-diethoxymethyl-1,2,3-triazole* (**a3b1**). Prepared from 72 µL (0.50 mmol) of **a3** and 73 mg (0.55 mmol) of **b1**. The product was obtained as a yellow solid (140 mg, 99%). C_14_H_19_N_3_O_2_, M = 261.33 g.mol^−1^. m.p. 60 ºC. FTIR: *ν* 3120, 3073, 2983, 2929, 2891, 1455, 1267, 1094, 1052 cm^−1^. ^1^H-NMR (CDCl_3_): *δ* 1.20 (t, *J* = 7.0 Hz, 6H), 3.62 (2q, *J* = 7.1 Hz, 4H), 5.50 (s, 2H), 5.68 (s, 1H), 7.25–7.35 (m, 5H), 7.49 (s, 1H) ppm. ^13^C-NMR (CDCl_3_): *δ* 15.1, 54.2, 61.6, 96.8, 121.8, 128.1, 128.7, 129.1, 134.5, 147.5 ppm. LC-MS: ELSD pur. 100%, UV pur. 100%; R_t_ = 8.35 min; *m/z*: 234 ([M+H-(CH_2_CH_2_)]^+^, 90%), 284 ([M+Na]^+^, 10%).

*1-Ethoxycarbonylmethyl-4-diethoxymethyl-1,2,3-triazole* (**a3b2**). Prepared from 72 µL (0.50 mmol) of **a3** and 71 mg (0.55 mmol) of **b2**. The product was obtained as a yellow oil (131 mg, 99%). C_11_H_19_N_3_O_4_, M = 257.29 g.mol^−1^. FTIR: *ν* 3136, 2987, 2883, 1750, 1218, 1064, 1048, 1023 cm^−1^. ^1^H-NMR (CDCl_3_): *δ* 1.12–1.22 (m, 9H), 3.57 (2q, *J* = 6.9 Hz, 4H), 4.51 (q, *J* = 7.2 Hz, 2H), 5.15 (s, 2H), 5.75 (s, 1H), 7.73 (s, 1H) ppm. ^13^C-NMR (CDCl_3_): *δ* 14.4, 15.5, 51.2, 61.8, 62.7, 97.0, 124.0, 148.1, 166.2 ppm. LC-MS: ELSD pur. 100%, UV pur. 100%; R_t_ = 5.33 min; *m/z*: 230 ([M+H-(CH_2_CH_2_)]^+^, 80%), 280 ([M+Na]^+^, 20%).

*4-Diethoxymethyl-1-(3-hydroxypropyl)-1,2,3-triazole* (**a3b3**). Prepared from 72 µL (0.50 mmol) of **a3** and 56 mg (0.55 mmol) of **b3**. The product was obtained as a yellow oil (119 mg, 99%). C_10_H_19_N_3_O_3_, M = 229.28 g.mol^−1^. FTIR: *ν* 3394, 2973, 2937, 2883, 1135, 1106, 1065 cm^−1^. ^1^H-NMR (CDCl_3_): *δ* 1.22 (t, *J* = 6.9 Hz, 6H), 2.12 (2q, *J* = 6.0 Hz, 2H), 2.74 (s, 1H), 3.56–3.70 (m, 6H), 4.51 (t, *J* = 6.0 Hz, 2H), 5.64 (s, 1H), 7.64 (s, 1H) ppm. ^13^C-NMR (CDCl_3_): *δ* 15.1, 32.6, 47.1, 58.4, 61.6, 96.8, 122.3, 147.0 ppm. LC-MS: ELSD pur. 100%, UV pur. 100%; R_t_ = 2.35 min; *m/z*: 202 ([M+H-(CH_2_CH_2_)]^+^, 70%), 252 ([M+Na]^+^, 30%).

*4-Diethoxymethyl-1-(3-trifluoroacetamidopropyl)-1,2,3-triazole* (**a3b4**). Prepared from 72 µL (0.50 mmol) of **a3** and 108 mg (0.55 mmol) of **b4**. The product was obtained as a yellow solid (182 mg, 99%). C_12_H_19_F_3_N_4_O_3_, M = 324.31 g.mol^−1^. m.p. 80 ºC. FTIR: *ν* 3219, 3078, 2974, 2937, 2895, 1717 1571, 1193, 1152, 1052 cm^−1^. ^1^H-NMR (CDCl_3_): *δ* 1.24 (t, *J* = 7.0 Hz, 6H), 2.24 (qn, *J* = 6.5 Hz, 2H), 3.44 (q, *J* = 6.4 Hz, 2H), 3.68 (2q, *J* = 7.4 Hz, 4H), 4.46 (t, *J* = 6.5 Hz, 2H), 5.69 (s, 1H), 7.65 (s, 1H) ppm. ^13^C-NMR (CDCl_3_): *δ* 15.1, 29.2, 37.1, 47.6, 61.8, 96.7, 113.9, 122.5, 147.0, 158.0 ppm. LC-MS: ELSD pur. 100%, UV pur. 100%; R_t_ = 5.45 min; *m/z*: 297 ([M+H-(CH_2_CH_2_)]^+^, 60%),347 ([M+Na]^+^, 40%).

*4-Diethoxymethyl-1-(2,2-dimethyl-1,3-dioxolan-4-yl)methyl-1,2,3-triazole* (**a3b5**). Prepared from 72 µL (0.50 mmol) of **a3** and 86 mg (0.55 mmol) of **b5**. The product was obtained as a yellow oil (97 mg, 68%). C_12_H_21_N_3_O_4_, M = 285.35 g.mol^−1^. FTIR: *ν* 3136, 2987, 2937, 2879, 1372, 1226, 1052 cm^−1^. ^1^H-NMR (CDCl_3_): *δ* 1.27 (t, *J* = 7.0 Hz, 6H), 1.34 (s, 6H), 3.62–3.79 (m, 5H), 4.14 (dd, *J* = 6.2 Hz, 8.8 Hz, 1H), 4.45–4.59 (m, 3H), 5.75 (s, 1H), 7.77 (s, 1H) ppm. ^13^C-NMR (CDCl_3_): *δ* 15.1, 25.2, 26.6, 52.2, 61.8, 66.4, 74.0, 96.9, 110.2, 123.3, 147.2 ppm. LC-MS: ELSD pur. 99%, UV pur. 100%; R_t_ = 5.32 min; *m/z*: 258 ([M+H-(CH_2_CH_2_)]^+^, 80%), 308 ([M+Na]^+^, 20%).

*1-[(Cyclohex-3-en-1-yl)methyl]-4-diethoxymethyl-1,2,3-triazole* (**a3b6**). Prepared from 72 µL (0.50 mmol) of **a3** and 75 mg (0.55 mmol) of **b6**. The product was obtained as a yellow oil (126 mg, 95%). C_14_H_23_N_3_O_2_, M = 265.36 g.mol^−1^. FTIR: *ν* 3144, 2983, 2933, 1375, 1106, 1035 cm^−1^. ^1^H-NMR (CDCl_3_): *δ* 1.24 (t, *J* = 7.0 Hz, 6H), 1.64–1.84 (m, 3H), 1.97–2.26 (m, 4H), 3.65 (2q, *J* = 7.1 Hz, 4H), 4.26 (d, *J* = 7.3 Hz, 2H), 5.63–5.29 (m, 2H), 5.71 (s, 1H), 7.57 (s, 1H) ppm. ^13^C-NMR (CDCl_3_): *δ* 15.2, 24.1, 25.8, 28.9, 34.7, 55.4, 61.7, 96.9, 122.2, 124.8, 127.1, 147.3 ppm. LC-MS: ELSD pur. 100% ; R_t_ = 8.76 min; *m/z*: 238 ([M+H-(CH_2_CH_2_)]^+^, 100%)

*4-Diethoxymethyl-1-[2-(2-thienyl)ethyl]-1,2,3-triazole* (**a3b7**). Prepared from 72 µL (0.50 mmol) of **a3** and 84 mg (0.55 mmol) of **b7**. The product was obtained as a yellow solid (101 mg, 72%). C_13_H_19_N_3_O_2_S, M = 281.38 g.mol^−1^. m.p. 53 ºC. FTIR: *ν* 3140, 3086, 2974, 2879, 1123, 1102, 1052 cm^−1^. ^1^H-NMR (CDCl_3_): *δ* 1.26 (t, *J* = 7.0 Hz, 6H), 3.47 (t, *J* = 7.1 Hz, 2H), 3.64 (2q, *J* = 7.1 Hz, 4H), 4.63 (t, *J* = 7.2 Hz, 2H), 5.73 (s, 1H), 6.76 (dd, *J* = 3.4, 0.7 Hz, 1H), 6.94 (dd, *J* = 3.4, 5.1 Hz, 1H), 7.21 (dd, *J* = 5.0, 1.1 Hz, 1H), 7.44 (s, 1H) ppm. ^13^C-NMR (CDCl_3_): *δ* 15.1, 30.8, 51.6, 61.5, 96.7, 122.3, 124.5, 126.1, 127.1, 138.7, 147.0 ppm. LC-MS: ELSD pur. 100%, UV pur. 100%; R_t_ = 8.42 min; *m/z*: 254 ([M+H-(CH_2_CH_2_)]^+^, 80%), 304 ([M+Na]^+^, 20%).

1-(3,6-Anhydro-1-deoxy-4,5-O-isopropylidene-D-glucitol-1-yl)-4-diethoxymethyl-1,2,3-triazole 

(**a3b8**). Prepared from 72 µL (0.50 mmol) of **a3** and 126 mg (0.55 mmol) of **b8**. The product was obtained as a yellow oil (172 mg, 95%). C_16_H_27_N_3_O_6,_ M = 357.41 g.mol^−1^. FTIR: *ν* 3455, 2956, 2913, 1375, 1195, 1103, 1057 cm^−1^. ^1^H-NMR (CDCl_3_): *δ* 1.24 (t, *J* = 7.1 Hz, 6H), 1.32 (s, 3H), 1.50 (s, 3H), 3.15 (dd, *J* = 3.6, 5.7 Hz, 1H), 3.43–3.57 (m, 3H), 3.66 (2q, *J* = 7.5 Hz, 4H), 4.07 (dd, *J* = 3.1, 10.8 Hz, 1H), 4.56 (dd, *J* = 3.5, 14.1 Hz, 1H), 4.64–4.73 (m, 1H), 4.77–4.85 (m, 2H), 5.73 (s, 1H), 7.80 (s, 1H) ppm. ^13^C-NMR (CDCl_3_): *δ* 15.1, 24.4, 25.9, 52.4, 61.6, 69.2, 72.6, 80.6, 81.3, 81.5, 96.8, 112.6, 123.9, 146.9 ppm. LC-MS: ELSD pur. 100%, UV pur. 100%; R_t_ = 3.27 min; *m/z*: 330 ([M+H-(CH_2_CH_2_)]^+^, 90%), 380 ([M+Na]^+^, 10%).

*1-Benzyl-4-methoxycarbonylmethyl-1,2,3-triazole* (**a4b1**). Prepared from 44 mg (0.50 mmol) of **a4** and 73 mg (0.55 mmol) of **b1**. The product (**a4b1**) was obtained as a off-white solid (107 mg, 99%). C_11_H_11_N_3_O_2_, M = 217.23 g.mol^−1^. m.p. 116–118 ºC. FTIR: *ν* 3112, 3066, 2950, 1725, 1538, 1239, 1048 cm^−1^. ^1^H-NMR (CDCl_3_): *δ* 3.90 (s, 3H), 5.55 (s, 2H), 7.27–7.36 (m, 5H), 7.99 (s, 1H) ppm. ^13^C-NMR (CDCl_3_): *δ* 52.2, 54.5, 127.3, 128.3, 129.2, 129.3, 133.6, 140.3, 161.1 ppm. LC-MS: ELSD pur. 89%, UV pur. 100%, R_t_ = 8.88 min, *m/z*: 218 ([M+H]^+^, 100%)

*1-Ethoxycarbonylmethyl-4-methoxycarbonylmethyl-1,2,3-triazole* (**a4b2**). Prepared from 44 mg (0.50 mmol) of **a4** and 71 mg (0.55 mmol) of **b2**.The product was obtained as a yellow solid (105 mg, 99%). C_8_H_11_N_3_O_4_, M = 213.19 g.mol^−1^. m.p. 104.5 ºC. FTIR: *ν* 3149, 3007, 2962, 1763, 1716, 1543, 1401, 1376, 1239, 1219, 1048, 1032 cm^−1^. ^1^H-NMR (CDCl_3_): *δ* 1.30 (t, *J* = 7.2 Hz, 3H), 3.95 (s, 3H), 4.28 (q, *J* = 7.2 Hz, 2H), 5.25 (s, 2H), 8.30 (s, 1H) ppm. ^13^C-NMR (CDCl_3_): *δ* 14.4, 51.4, 52.7, 63.2, 129.4, 161.4, 166.0 ppm. LC-MS: ELSD pur. 91%, UV pur. 100%, R_t_ = 3.68 min, *m/z*: 214 ([M+H]^+^, 100%).

*1-(3-Hydroxypropyl)-4-methoxycarbonylmethyl-1,2,3-triazole* (**a4b3**). Prepared from 44 mg (0.50 mmol) of **a4** and 56 mg (0.55 mmol) of **b3**. The product was obtained as an orange solid (119 mg, 76%). C_7_H_11_N_3_O_3_, M = 185.18 g.mol^−1^. m.p. 52 ºC. FTIR: *ν* 3124, 2958, 2875, 1737, 1721, 1543, 1223, 1044 cm^−1^. ^1^H-NMR (CDCl_3_): *δ* 2.18 (q, *J* = 6.0 Hz, 2H), 3.67 (t, *J* = 6.0 Hz, 2H), 3.93 (s, 3H), 4.62 (t, *J* = 6.0 Hz, 2H), 8.22 (s, 1H) ppm. ^13^C-NMR (CDCl_3_): *δ* 32.4, 47.5, 52.2, 58.3, 128.2, 139.7, 161.2 ppm. LC-MS: ELSD pur. 99%, UV pur. 100%, R_t_ = 2.98 min, *m/z*: 186 ([M+H]^+^, 100%).

*1-(3-Trifluoroacetamidopropyl)-4-methoxycarbonylmethyl-1,2,3-triazole* (**a4b4**). Prepared from 44 mg (0.50 mmol) of **a4** and 108 mg (0.55 mmol) of **b4**. The product was obtained as an orange solid (125 mg, 89%). C_9_H_11_F_3_N_4_O_3_, M = 280.21 g.mol^−1^. m.p. 67 ºC. FTIR: *ν* 3290, 3137, 3095, 2962, 1563, 1219, 1194, 1160, 1048 cm^−1^. ^1^H-NMR (CDCl_3_): *δ* 1.90 (t, *J* = 6.5 Hz, 3H), 3.48 (q, *J* = 6.2 Hz, 2H), 3.98 (s, 2H), 4.54 (t, *J* = 6.6 Hz, 2H), 6.86 (s, 1H), 8.24 (s, 1H) ppm. ^13^C-NMR (CDCl_3_): *δ* 28.0, 37.7, 49.2, 50.6, 126.2, 128.1, 139.9, 161,1 ppm. LC-MS: ELSD pur. 61%, UV pur. 100%, R_t_ = 3.32 min, *m/z*: 181 ([M+H]^+^, 100%).

*4-Methoxycarbonylmethyl-1-(2,2-dimethyl-1,3-dioxolan-4-yl)methyl-1,2,3-triazole* (**a4b5**). Prepared from 44 mg (0.50 mmol) of **a4** and 86 mg (0.55 mmol) of **b5**. The product was obtained as a yellow solid (89 mg, 89%). C_10_H_15_N_3_O_4_, M = 241.25 g.mol^−1^. m.p. 103 ºC. FTIR: *ν* 3120, 2987, 2953, 2863, 1721, 1547, 1252, 1044 cm^−1^. ^1^H-NMR (CDCl_3_): *δ* 1.32 (s, 3H), 1.37 (s, 3H), 3.73 (dd, *J* = 5.3, 8.7 Hz, 1H), 4.10 (dd, *J* = 6.1, 8.7 Hz, 1H), 4.36–4.56 (m, 3H), 4.74 (s, 2H), 7.73 (s, 1H) ppm. ^13^C-NMR (CDCl_3_): *δ* 25.1, 26.6, 52.2, 53.1, 66.2, 73.7, 110.4, 128.8, 139.9, 161.1 ppm. LC-MS: ELSD pur. 95%, UV pur. 100%, R_t_ = 2.18 min, *m/z*: 214 ([M+H]^+^, 100%).

*1-[(Cyclohex-3-en-1-yl)methyl]-4-methoxycarbonylmethyl-1,2,3-triazole* (**a4b6**). Prepared from 44 mg (0.50 mmol) of **a4** and 75 mg (0.55 mmol) of **b6**. The product was obtained as a grey solid (96,7 mg, 82%). C_11_H_15_N_3_O_2_, M = 221.26 g.mol^−1^. m.p. 94 ºC. FTIR: *ν* 3124, 3016, 2921, 2845, 1721, 1547, 1235, 1040 cm^−1^. ^1^H-NMR (CDCl_3_): *δ* 1.32 (m, 2H), 1.75 (m, 2H), 2.02 (m, 2H), 2.20 (m, 1H), 4.27 (d, *J* = 7.3 Hz, 2H), 4.78 (s, 2H), 5.60–5.72 (m, 2H), 7.58 (s, 1H) ppm. ^13^C-NMR (CDCl_3_): *δ* 24.0, 25.7, 28.7, 34.7, 52.2, 55.7, 124.6, 127.1, 161.3 ppm. LC-MS: ELSD pur. 80%, UV pur. 100%, R_t_ = 3.06 min, *m/z*: 194 ([M+H]^+^, 100%).

*4-Methoxycarbonylmethyl-1-[2-(2-thienyl)ethyl]-1,2,3-triazole* (**a4b7**). Prepared from 44 mg (0.50 mmol) of **a4** and 84 mg (0.55 mmol) of **b7**. The product was obtained as a yellow solid (61 mg, 58%). C_10_H_11_N_3_O_2_S, M = 237.28 g.mol^−1^. m.p. 136.5 ºC. FTIR: *ν* 3095, 3037, 2953, 2921, 2850, 1729, 1534, 1231, 1053 cm^−1^. ^1^H-NMR (CDCl_3_): *δ* 3.42 (t, *J* = 7.1 Hz, 2H), 4.59 (t, *J* = 7.1 Hz, 2H), 4.73 (s, 2H), 6.74 (d, *J* = 3.3 Hz, 1H), 6.91 (dd, *J* = 3.3, 5.0 Hz, 1H), 7.16 (d, *J* = 5.1 Hz, 1H), 7.47 (s, 1H) ppm. ^13^C-NMR (CDCl_3_): *δ* 30.6, 52.1, 124.9, 126.4, 127.3, 127.9, 138.2, 139.7, 161.1 ppm. LC-MS: ELSD pur. 95%, UV pur. 100%, R_t_ = 2.74 min, *m/z*: 210 ([M+H]^+^, 100%).

*1-(3,6-Anhydro-1-deoxy-4,5-O-isopropylidene-D-glucitol-1-yl)-4-methoxycarbonylmethyl-1,2,3-triazole* (**a4b8**). Prepared from 44 mg (0.50 mmol) of **a4** and 126 mg (0.55 mmol) of **b8**. The product was obtained as a pale yellow oil (155,1 mg, 99%). C_13_H_19_N_3_O_6_, M = 313.31 g.mol^−1^. FTIR: *ν* 3445, 3130, 2979, 2854, 1736, 1542, 1437, 1275, 1208, 1099, 1068 cm^−1^. ^1^H-NMR (CDCl_3_): *δ* 1.33 (s, 3H), 1.50 (s, 3H), 3.13 (dd, *J* = 3.5, 5.5 Hz, 1H), 3.46 (dd, *J* = 3.5, 10.8 Hz, 1H), 3.94 (s, 3H), 4.07 (d, *J* = 10.8 Hz, 1H), 4.40–4.44 (m, 1H), 4.64 (dd, *J* = 3.4, 14.2 Hz, 1H), 4.74 (dd, *J* = 5.6, 14.2 Hz, 1H), 4.79–4.87 (m, 2H), 8.33 (s, 1H) ppm. ^13^C-NMR (CDCl_3_): *δ* 24.3, 25.9, 52.2, 52.9, 69.0, 72.7, 80.7, 81.3, 112.8, 129.5, 161.3 ppm. LC-MS: ELSD pur. 95%, UV pur. 100%, R_t_ = 2.74 min, *m/z*: 210 ([M+H]^+^, 100%).

*1-Benzyl-4-phthalimidomethyl-1,2,3-triazole* (**a5b1**). Prepared from 93 mg (0.50 mmol) of **a5** and 73 mg (0.55 mmol) of **b1**. The product was obtained as a white solid (148 mg, 93%). C_18_H_14_N_4_O_2_, M = 318.34 g.mol^−1^. m.p. 179–181 ºC. FTIR: *ν* 3110, 3076, 3038, 2849, 1771, 1708, 1432, 1402, 1097 cm^−1^. ^1^H-NMR (CDCl_3_): *δ* 4.97 (s, 2H), 5.49 (s, 2H), 7.25–7.37 (m, 5H), 7.51 (s, 1H), 7.70–7.85 (m, 4H) ppm. ^13^C-NMR (CDCl_3_): *δ* 33.1, 54.2, 122.7, 123.4, 128.1, 128.7, 129.1, 132.0, 134.1, 134.5, 143.1, 167.6 ppm. LC-MS: ELSD pur. 98%, UV pur. 100%; R_t_ = 9.10 min; *m/z*: 319 ([M+H]^+^).

*1-Ethoxycarbonylmethyl-4-phthalimidomethyl-1,2,3-triazole* (**a5b2**). Prepared from 93 mg (0.50 mmol) of **a5** and 71 mg (0.55 mmol) of **b2**. The product was obtained as a white solid (145 mg, 92%). C_15_H_14_N_4_O_4_. M =314.30 g.mol^−1^. m.p. 106–108 ºC. FTIR: *ν* 3122, 3066, 2998, 2955, 2888, 1755, 1701, 1446, 1402, 1210, 1102, 1052 cm^−1^. ^1^H-NMR (CDCl_3_): *δ* 1.27 (t, *J* = 7.2 Hz, 3H), 4.23 (q, *J* = 7.2 Hz, 2H), 4.99 (s, 2H), 5.11 (s, 2H), 7.70–7.82 (m, 5H) ppm. ^13^C-NMR (CDCl_3_): *δ* 14.0 5, 32.9, 50.8, 62.3, 123.4, 124.4, 131.9, 134.1, 143.0, 166.2, 167.6 ppm. LC-MS: ELSD pur. 99%, UV pur. 100%; R_t_ = 5.63 min; *m/z*: 315 ([M+H]^+^).

*1-(3-Hydroxypropyl)-4-phthalimidomethyl-1,2,3-triazole* (**a5b3**). Prepared from 93 mg (0.50 mmol) of **a5** and 56 mg (0.55 mmol) of **b3**. The product was obtained as a white solid (130 mg, 90%). C_14_H_14_N_4_O_3_, M = 286.29 g.mol^−1^. m.p. 108–109 ºC. FTIR: *ν* 3316, 3128, 2937, 2863, 1766, 1697, 1421, 1397, 1092, 1052 cm^−1^. ^1^H-NMR (CDCl_3_): *δ* 2.06 (q, *J =* 6.0 Hz, 2H), 3.61 (t, *J =* 6.0 Hz, 2H), 4.47 (t, *J =* 6.0 Hz, 2H), 4.96 (s, 2H), 7.68–7.71 (m, 2H), 7.81–7.83 (m, 2H) ppm. ^13^C-NMR (CDCl_3_): *δ* 32.5, 33.0, 46.9, 58.8, 123.2, 123.4, 132.0, 134.1, 167.7 ppm. LC-MS: ELSD pur. 99%, UV pur. 100%; R_t_ = 8.23 min; *m/z*: 287 ([M+H]^+^).

*1-(3-Trifluoroacetamidopropyl)-4-phthalimidomethyl-1,2,3-triazole* (**a5b4**). Prepared from 93 mg (0.50 mmol) of **a5** and 108 mg (0.55 mmol) of **b4**. The product was obtained as a white solid (107 mg, 57%). C_16_H_14_F_3_N_5_O_3_, M = 381.32 g.mol^−1^. m.p. 171–173 ºC. FTIR: *ν* 3350, 3150, 2094, 2961, 2872, 1766, 1712, 1559, 1402, 1427, 1219, 1186, 1141, 1048 cm^−1^. ^1^H-NMR (CDCl_3_): *δ* 2.18 (q, *J =* 6.0 Hz, 2H), 3.36 (t, *J =* 6.0 Hz, 2H), 4.41 (t, *J =* 6.0 Hz, 2H), 4.99 (s, 2H), 7.75–7.66 (m, 2H), 7.88–7.83 (m, 2H) ppm. ^13^C-NMR (CDCl_3_): *δ* 28.5, 32.6, 36.3, 47.0, 122.7, 131.7, 133.9, 167.4 ppm. LC-MS: ELSD pur. 91%, UV pur. 100%; R_t_ = 5.81 min; *m/z*: 382 ([M+H]^+^).

*1-(2,2-Dimethyl-1,3-dioxolan-4-yl)methyl-4-phthalimidomethyl-1,2,3-triazole* (**a5b5**). Prepared from 93 mg (0.50 mmol) of **a5** and 86 mg (0.55 mmol) of **b5**. The product was obtained as a white solid (170 mg, 99%). C_17_H_18_N_4_O_4_, M = 342.36 g.mol^−1^. m.p.147–149 ºC. FTIR: *ν* 3124, 3074, 2981, 2941, 2873, 1771, 1706, 1431, 1402, 1102, 1072, 1043 cm^−1^. ^1^H-NMR (CDCl_3_): *δ* 1.33 (s, 3H), 1.34 (s, 3H), 3.73 (dd, *J =* 9.6 Hz, 1H), 4.11 (dd, *J =* 9.6 Hz, 1H), 4.54–4.39 (m, 3H), 5.00 (s, 2H), 7.75–7.71 (m, 2H), 7.76 (s, 1H), 7.86–7.83 (m, 2H) ppm. ^13^C-NMR (CDCl_3_): *δ* 25.6, 27.0, 33.4, 52.6, 66.8, 74.3, 110.5, 123.8, 124.5, 132.5, 134.4, 143.1, 167.9 ppm. LC-MS: ELSD pur. 99%, UV pur. 100%; R_t_ = 5.38 min; *m/z*: 343 ([M+H]^+^).

*1-[(Cyclohex-3-en-1-yl)methyl]-4-phthalimidomethyl-1,2,3-triazole* (**a5b6**). Prepared from 93 mg (0.50 mmol) of **a5** and 75 mg (0.55 mmol) of **b6**. The product was obtained as a beige solid (165 mg, 99%). C_18_H_18_N_4_O_2_, M =322.37 g.mol^−1^. m.p.135–137 ºC. FTIR: *ν* 3128, 3074, 3025, 2927, 2843, 1771, 1701, 1427, 1397, 1210, 1097 cm^−1^. ^1^H-NMR (CDCl_3_): *δ* 1.34–1.25 (m, 1H), 2.22–1.66 (m, 6H), 4.23 (d, *J =* 7.3 Hz, 2H), 5.04 (s, 2H), 5.70–5.65 (m, 2H) 7.62 (s, 1H), 7.77–7.74 (m, 2H), 7.91–7.88 (m, 2H) ppm. ^13^C-NMR (CDCl_3_): *δ* 24.1, 25.8, 28.9, 33.1, 34.6, 55.4, 123.4, 124.8, 127.0, 132.1, 134.1, 142.7, 167.6 ppm. LC-MS: ELSD pur. 99%, UV pur. 100%; R_t_ = 8.60 min; *m/z*: 323 ([M+H]^+^).

*4-Phthalimidomethyl-1-[2-(2-thienyl)ethyl]-1,2,3-triazole* (**a5b7**). Prepared from 93 mg (0.50 mmol) of **a5** and 84 mg (0.55 mmol) of **b7**. The product was obtained as a white solid (138 mg, 82%). C_17_H_14_N_4_O_2_S, M = 338.39 g.mol^−1^. m.p.149–151 ºC. FTIR: *ν* 3128, 3084, 2957, 2927, 1766, 1712, 1427, 1397, 1092 cm^−1^. ^1^H-NMR (CDCl_3_): *δ* 3.43 (t, *J =* 7 Hz, 2H), 4.60 (t, *J =* 7.1 Hz, 2H), 5.00 (s, 2H), 6.73 (dd, *J =* 3.4, 0.9 Hz, 1H), 6.91 (dd, *J =* 5.1, 3.5 Hz, 1H), 7.17 (dd, *J =* 5.1, 1.1 Hz, 1H), 7.46 (s, 1H), 7.78–7.76 (m, 2H), 7.90–7.88 (m, 2H) ppm. ^13^C-NMR (CDCl_3_): *δ* 31.1, 33.4, 52.0, 79.62, 123.7, 124.8, 126.5, 127.5, 132.5, 134.4, 139.1, 143.0, 167.9 ppm. LC-MS: ELSD pur. 99%, UV pur. 100%; R_t_ = 8.20 min; *m/z*: 339 ([M+H]^+^).

*1-(3,6-Anhydro-1-deoxy-4,5-O-isopropylidene-D-glucitol-1-yl)-4-phthalimidomethyl-1,2,3-triazole* (**a5b8**). Prepared from 93 mg (0.50 mmol) of **a5** and 126 mg (0.55 mmol) of **b8**. The product was obtained as a white solid (205 mg, 99%). C_20_H_22_N_4_O_6_, M = 414.42 g.mol^−1^. m.p.189–191 ºC. FTIR: *ν* 3439, 3149, 2971, 2932, 2853, 1771, 1717, 1427, 1402, 1102, 1052 cm^−1^. ^1^H-NMR (CDCl_3_): *δ* 1.31 (s, 3H), 1.34 (s, 3H), 3.09–3.14 (m, 3H), 3.45 (dd, *J =* 10.7, 3.5 Hz, 1H), 4.07 (d, *J =* 10.7 Hz, 1H), 4.37–4.36 (m,1H), 4.54 (dd, *J =* 14.2, 3.5 Hz, 1H), 4.67 (dd, *J =* 14.4, 5.6 Hz, 1H), 4.83–4.75 (m, 2H), 4.99 (s, 1H) 7.73–7.70(m, 2H) 7.74 (s, 1H), 7.87–7.84 (m, 2H) ppm. ^13^C-NMR (CDCl_3_): *δ* 24.4, 25.9, 33.2, 52.5, 69.2, 72.7, 80.6, 81.4, 81.7, 112.6, 123.5, 124.7, 132.2, 134.1, 142,6, 167.9 ppm. LC-MS: ELSD pur. 97%, UV pur. 100%; R_t_ = 3.54 min; *m/z*: 415 ([M+H]^+^).

*4-Acetamidomethyl-1-benzyl-1,2,3-triazole* (**a6b1**). Prepared from 49 mg (0.50 mmol) of **a6** and 73 mg (0.55 mmol) of **b1**. The product was obtained as a beige solid (96 mg, 83%). C_12_H_14_N_4_O, M = 230.27 g.mol^−1^. m.p. 118 ºC. FTIR: *ν* 3248, 3132, 3073, 2941, 1651, 1534, 1426, 1293 cm^−1^. ^1^H-NMR (CDCl_3_): *δ* 1.98 (s, 3H), 4.47 (d, *J* = 5.4 Hz, 2H), 5.49 (s, 2H), 5.51 (s, 2H), 6.18 (br s, 1H), 7.25–7.38 (m,15H), 7.47 (s, 1H) ppm. ^13^C-NMR (CDCl_3_): *δ* 23.1, 34.9, 54.3, 128.2, 128.9, 129.2, 134.4, 170.1 ppm. LC-MS: ELSD pur. 100%, UV pur. 100%; R_t_ = 2.86 min; *m/z*: 231 ([M+H]^+^, 50%), 253 ([M+Na]^+^, 25%), 294 ([M+Cu]^+^, 25%).

*4-Acetamidomethyl-1-ethoxycarbonylmethyl-1,2,3-triazole* (**a6b2**). Prepared from 49 mg (0.50 mmol) of **a6** and 71 mg (0.55 mmol) of **b2**. The product was obtained as a beige solid (83 mg, 73%). C_9_H_14_N_4_O_3_, M = 226.24 g.mol^−1^. m.p. 102 ºC. FTIR: *ν* 3252, 3140, 3066, 3003, 2970, 1746, 1671, 1550, 1218 cm^−1^. ^1^H-NMR (CDCl_3_): *δ* 1.31 (t, *J* = 7.2 Hz, 3H), 1.99 (s, 3H), 4.27 (q, *J* = 7.2 Hz, 2H), 4.52 (d, *J* = 5.4 Hz, 2H), 5.14 (s, 2H), 6.53 (br s, 1H), 7.70 (s, 1H) ppm. ^13^C-NMR (CDCl_3_): *δ* 14.0, 23.1, 34.9, 50.9, 362.5, 123.9, 166.2, 170.2 ppm. LC-MS: ELSD pur. 100%, UV pur. 100%; R_t_ = 2.23 min; *m/z*: 227 ([M+H]^+^, 70%), 249 ([M+Na]^+^, 30%).

*4-Acetamidomethyl-1-(3-hydroxypropyl)-1,2,3-triazole* (**a6b3**). Prepared from 49 mg (0.50 mmol) of **a6** and 56 mg (0.55 mmol) of **b3**. The product was obtained as a yellow oil (42 mg, 47%). C_8_H_14_N_4_O_2_, M = 198.23 g.mol^−1^. FTIR: *ν* 3283, 3089, 2924, 2875, 1634, 1552, 1440, 1288, 1052 cm^−1^. ^1^H-NMR (CDCl_3_): *δ* 1.99 (s, 3H), 2.08–2.16 (m, 2H), 3.63 (t, *J* = 5.4 Hz, 2H), 4.48–4.53 (m, 4H), 6.84 (br s, 1H), 7.67 (s, 1H) ppm. ^13^C-NMR (CDCl_3_): *δ* 23.1, 32.5, 34.8, 47.1, 58.5, 123.1, 170.5 ppm. LC-MS: ELSD pur. 100%, UV pur. 100%; R_t_ = 1.67 min; *m/z*: 199 ([M+H]^+^, 75%), 221 ([M+Na]^+^, 25%).

*4-Acetamidomethyl-1-(3-trifluoroacetamidopropyl)-1,2,3-triazole* (**a6b4**). Prepared from 49 mg (0.50 mmol) of **a6** and 108 mg (0.55 mmol) of **b4**. The product was obtained as a beige solid (107 mg, 73%). C_10_H_14_F_3_N_5_O_2_, M = 293.25 g.mol^−1^. m.p. 135 ºC. FTIR: *ν* 3277, 3232, 3086, 2953, 1728, 1650, 1559, 1202, 1148 cm^−1^. ^1^H-NMR (CDCl_3_): *δ* 1.87 (s, 3H), 2.04–2.11 (m, 2H), 3.20–3.24 (m, 2H), 4,30–4.35 (m, 4H), 7.79 (s, 1H) ppm. ^13^C-NMR (CDCl_3_): *δ* 22.4, 30.4, 35.7, 37.9 123.6, 173.3 ppm. LC-MS: ELSD pur. 100%, UV pur. 100%; R_t_ = 2.22 min; *m/z*: 294 ([M+H]^+^, 60%), 316 ([M+Na]^+^, 40%).

*4-Acetamidomethyl-1-(2,2-dimethyl-1,3-dioxolan-4-yl)methyl-1,2,3-triazole* (**a6b5**). Prepared from 49 mg (0.50 mmol) of **a6** and 86 mg (0.55 mmol) of **b5**. The product was obtained as a yellow solid (110 mg, 87%). C_10_H_16_N_4_O_3_, M = 240.26 g.mol^−1^. m.p. 87 ºC. FTIR: *ν* 3269, 3078, 2999, 2929, 1629, 1546, 1375, 1210, 1056 cm^−1^. ^1^H-NMR (CDCl_3_): *δ* 1.34 (s, 3H), 1.38 (s, 3H), 3.74 (dd, *J* = 5.7, 9 Hz, 1H), 4.12 (dd, *J* = 6.3, 8.7 Hz, 1H), 4.37–4.57 (m, 5H), 6.53 (br s, 1H), 7.70 (s, 1H) ppm. ^13^C-NMR (CDCl_3_): *δ* 23.1, 25.1, 26.7, 34.8, 52.5, 66.4, 73.9, 110.3, 123.6, 170.1 ppm. LC-MS: ELSD pur. 100%, UV pur. 100%; R_t_ = 2.08 min; *m/z*: 255 ([M+H]^+^, 60%), 277 ([M+Na]^+^, 30%), 318 ([M+Cu]^+^, 10%).

*4-Acetamidomethyl-1-[(cyclohex-3-en-1-yl)methyl]-1,2,3-triazole* (**a6b6**). Prepared from 49 mg (0.50 mmol) of **a6** and 75 mg (0.55 mmol) of **b6**. The product was obtained as a brown solid (103 mg, 88%). C_12_H_18_N_4_O, M = 234.30 g.mol^−1^. FTIR: *ν* 3244, 3140, 3057, 2929, 2850, 1658, 1566, 1289 cm^−1^. ^1^H-NMR (CDCl_3_): *δ* 1.18–1.25 (m, 1H), 1.65–1.75 (m, 2H), 1.93 (s, 3H), 1.96–2.10 (m, 4H), 4.18 (d, *J* = 7.2 Hz, 2H), 4.40 (d, *J* = 5.7 Hz, 2H), 5.56–5.62 (m, 2H), 6.49 (br s, 1H), 7.47 (s, 1H) ppm. ^13^C-NMR (CDCl_3_): *δ* 22.1, 23.1, 24.8, 27.9, 33.6, 84.5, 123.7, 126.1, 170.1 ppm. LC-MS: ELSD pur. 100% ; R_t_ = 2.92 min; *m/z*: 235 ([M+H]^+^, 50%), 257 ([M+Na]^+^, 30%), 298 ([M+Cu]^+^, 20%).

*4-Acetamidomethyl-1-[2-(2-thienyl)ethyl]-1,2,3-triazole* (**a6b7**). Prepared from 49 mg (0.50 mmol) of **a6** and 84 mg (0.55 mmol) of **b7**. The product was obtained as a beige solid (108 mg, 86%). C_11_H_14_N_4_OS, M = 250.32 g.mol^−1^. m.p. 117 ºC. FTIR: *ν* 3264, 3140, 3078, 2953, 2862, 1654, 1550, 1438, 1289 cm^−1^. ^1^H-NMR (CDCl_3_): *δ* 1.99 (s, 3H), 3.42 (t, *J* = 6.9 Hz, 2H), 4.46 (d, *J* = 5.4 Hz, 2H), 4.58 (t, *J* = 7.2 Hz, 2H), 6.49 (br s, 1H), 6.73–6.74 (m, 1H), 6.90–6.93 (m, 1H), 7.17–7.18 (m, 1H), 7.41 (s, 1H) ppm. ^13^C-NMR (CDCl_3_): *δ* 23.1, 30.8, 34.9, 51.7, 123.4, 124.6, 126.1, 127.2, 138.7, 170.1 ppm. LC-MS: ELSD pur. 100%, UV pur. 100%; R_t_ = 2.46 min; *m/z*: 251 ([M+H]^+^, 50%), 273 ([M+Na]^+^, 30%), 314 ([M+Cu]^+^, 20%).

*4-Acetamidomethyl-1-(3,6-anhydro-1-deoxy-4,5-O-isopropylidene-D-glucitol-1-yl)-1,2,3-triazole* (**a6b8**). Prepared from 49 mg (0.50 mmol) of **a6** and 126 mg (0.55 mmol) of **b8**. The product was obtained as a yellow oil (87 mg, 53%). C_14_H_22_N_4_O_5_, M = 326.36 g.mol^−1^. FTIR: *ν* 3356, 3235, 3127, 3073, 2983, 2945, 2850, 1658, 1546, 1384, 1210, 1106 cm^−1^. ^1^H-NMR (CDCl_3_): *δ* 1.33 (s, 3H), 1.50 (s, 3H), 2.00 (s, 3H), 3.18 (dd, *J* = 3.3, 6.0 Hz, 1H), 3.44–3.54 (m, 3H), 4.07 (d, *J* = 12.4 Hz, 1H), 4.50 (d, *J* = 4.8 Hz, 2H), 4.56 (dd, *J* = 3.6, 14.2 Hz, 1H), 4.65 (dd, *J* = 6.0, 14.2 Hz, 1H), 4.78–4.85 (m, 2H), 6.45 (br s, 1H), 7.73 (s, 1H) ppm. ^13^C-NMR (CDCl_3_): *δ* 23.1, 24.3, 25.8, 34.9, 52.6, 69.2, 72.6, 80.6, 81.3, 81.5, 112.6, 124.0, 170.2 ppm. LC-MS: ELSD pur. 100%, UV pur. 100%; R_t_ = 2.23 min; *m/z*: 327 ([M+H]^+^, 60%), 349 ([M+Na]^+^, 40%).

*1-Benzyl-4-trifluoroacetamidomethyl-1,2,3-triazole* (**a7b1**). Prepared from 76 mg (0.50 mmol) of **a7** and 73 mg (0.55 mmol) of **b1**. The product was obtained as a white solid (140 mg, 98%). C_12_H_11_F_3_N_4_O, M = 284.24 g.mol^−1^. m.p. 132–134 ºC. FTIR: *ν* 3302, 3107, 3061, 1699, 1563, 1210, 1185, 1156, 1060 cm^−1^. ^1^H-NMR (CDCl_3_): *δ* 4.58 (d, *J =* 4.67 Hz, 2H), 5.51 (s, 2H), 7.46–7.20 (m, 5H), 7.54 (s, 1H), 7.70 (s, 1H) ppm. ^13^C-NMR (CDCl_3_): *δ* 28.3, 36.2, 54.5, 128.2, 129.03, 129.3, 134.1, 157.1 ppm. LC-MS: ELSD pur. 99%, UV pur. 100%; R_t_ = 7.37 min; *m/z*: 285 ([M+H]^+^).

*1-Ethoxycarbonylmethyl-4-trifluoroacetamidomethyl-1,2,3-triazole* (**a7b2**). Prepared from 76 mg (0.50 mmol) of **a7** and 71 mg (0.55 mmol) of **b2**. The product was obtained as a white solid (139 mg, 99%). C_9_H_11_F_3_N_4_O_3_, M = 280.21 g.mol^−1^. m.p. 140–142 ºC. FTIR: *ν* 3153, 3045, 3003, 2958, 2895, 1741, 1699, 1546, 1193, 1148, 1060 cm^−1^. ^1^H-NMR (CDCl_3_) : *δ* 1.31 (t, *J =* 7.1 Hz, 3H), 4.28 (q, *J =* 7.1 Hz, 2H), 4.62 (d, *J =* 5.7 Hz, 2H), 5.16 (s, 2H), 7.76 (s, 1H), 8.12 (s, 1H) ppm. ^13^C-NMR (CDCl_3_): *δ* 14.0, 35.1, 50.9, 62.6, 124.2, 166.4 ppm. LC-MS: ELSD pur. 99%, UV pur. 100%; R_t_ = 2.92 min; *m/z*: 281 ([M+H]^+^).

*4-Trifluoroacetamidomethyl-1-(3-hydroxypropyl)-1,2,3-triazole* (**a7b3**). Prepared from 76 mg (0.50 mmol) of **a7** and 56 mg (0.55 mmol) of **b3**. The product was obtained as a white solid (109 mg, 87%). C_8_H_11_F_3_N_4_O_2_, M = 252.20 g.mol^−1^. m.p. 84–86 ºC. FTIR: *ν* 3306, 3127, 3045, 3003, 1699, 1550, 1355, 1189, 1156, 1048 cm^−1^. ^1^H-NMR (CDCl_3_): *δ* 2.12 (q, *J* = 5.1 Hz, 2H), 3.62 (m, 2H), 4.52 (t, *J =* 6.8 Hz, 2H), 4.59 (d, *J =* 5.6 Hz, 2H), 4.74 (s, 1H), 7.67 (s, 1H), 8.10 (s, 1H) ppm. ^13^C-NMR (CDCl_3_): *δ* 32.5, 35.1, 47.1, 58.4, 87.9, 159.7 ppm. LC-MS: ELSD pur. 99%, UV pur. 100%; R_t_ = 2.22 min; *m/z*: 253 ([M+H]^+^).

*4-Trifluoroacetamidomethyl-1-(3-trifluoroacetamidopropyl)-1,2,3-triazole* (**a7b4**). Prepared from 76 mg (0.50 mmol) of **a7** and 108 mg (0.55 mmol) of **b4**. The product was obtained as a white solid (152 mg, 88%). C_10_H_11_F_6_N_5_O_2_, M = 347.22 g.mol^−1^. m.p. 120–122 ºC. FTIR: *ν* 3298, 3140, 2899, 1699, 1566, 1181, 1148, 1047 cm^−1^. ^1^H-NMR (CDCl_3_): *δ* 2.04 (p, *J =* 6.9 Hz, 2H), 3.20 (q, *J =* 6.7 Hz, 2H), 4.29 (t, *J =* 6.9 Hz, 2H), 4,43 (d, *J =* 5.6, 2H), 7.53 (s, 1H), 7.66 (s, 1H), 7.92 (s, 1H) ppm. ^13^C-NMR (CDCl_3_): *δ* 28.6, 34.5, 36.4, 47.1, 113.8, 122.8, 156.3 ppm. LC-MS: ELSD pur. 99%, UV pur. 100%; R_t_ = 4.02 min; *m/z*: 348 ([M+H]^+^).

*4-Trifluoroacetamidomethyl-1-(2,2-dimethyl-1,3-dioxolan-4-yl)methyl-1,2,3-triazole* (**a7b5**). Prepared from 76 mg (0.50 mmol) of **a7** and 86 mg (0.55 mmol) of **b5**. The product was obtained as a pale yellow solid (138 mg, 89%). C_11_H_15_F_3_N_4_O_3_, M = 308.26 g.mol^−1^. m.p. 126–128 ºC. FTIR: *ν* 3149, 3057, 2991, 1708, 1555, 1428, 1202, 1185, 1152, 1064 cm^−1^. ^1^H-NMR (CDCl_3_): *δ* 1.34 (s, 3H), 1.37 (s, 3H), 3.74 (dd, *J =* 8.8, 5.6 Hz, 1H), 4.13 (dd, *J =* 8.8, 6.2 Hz, 1H), 4.51–4.34 (m, 1H), 4.74–4.52 (m, 3H), 7.79 (s, 1H), 7.99 (s, 1H) ppm. ^13^C-NMR (CDCl_3_): *δ* 25.1, 26.6, 34.9, 52.6, 66.3, 73.9, 110.4, 117.7, 124.4, 157.7 ppm. LC-MS: ELSD pur. 99%, UV pur. 100%; R_t_ = 3.67 min; *m/z*: 309 ([M+H]^+^).

*1-[(Cyclohex-3-en-1-yl)methyl]-4-trifluoroacetamidomethyl-1,2,3-triazole* (**a7b6**). Prepared from 76 mg (0.50 mmol) of **a7** and 75 mg (0.55 mmol) of **b6**. The product was obtained as a beige solid (130 mg, 90%). C_12_H_15_F_3_N_4_O, M = 288.27 g.mol^−1^. m.p. 124–126 ºC . FTIR: *ν* 3156, 3032, 2899, 1721, 1566, 1429, 1193, 1152, 1064 cm^−1^. ^1^H-NMR (CDCl_3_): *δ* 1.45–1.19 (m, 1H), 1.88–1.61 (m, 2H), 2.20–1.91 (m, 4H), 4.27 (d, *J =* 7.2 Hz, 2H), 4.59 (d, *J =* 5.5 Hz, 2H), 5.62 (dt, *J =* 9.9, 2.1 Hz, 1H), 5.70 (dd, *J =* 10.0, 1.6 Hz, 1H), 7.61 (s, 1H), 8.35 (s, 1H) ppm. ^13^C-NMR (CDCl_3_): *δ* 24.1, 25.8, 28.8, 34.6, 35.0, 55.5, 123.2, 124.7, 127.1, 157.8 ppm. LC-MS: ELSD pur. 99%, UV pur. 100%; R_t_ = 8.07 min; *m/z*: 289 ([M+H]^+^).

*4-Trifluoroacetamidomethyl-1-[2-(2-thienyl)ethyl]-1,2,3-triazole* (**a7b7**). Prepared from 76 mg (0.50 mmol) of **a7** and 84 mg (0.55 mmol) of **b7**. The product was obtained as a beige solid (150 mg, 98%). C_11_H_11_F_3_N_4_OS, M = 304.30 g.mol^−1^. m.p. 108–110 ºC. FTIR: *ν* 3306, 3149, 3086, 2958, 1692, 1546, 1339, 1206, 1177, 1156, 1052 cm^−1^. ^1^H-NMR (CDCl_3_) : *δ* 3.43 (t, *J =* 7 Hz, 2H), 4.69–4.46 (m, 4H), 6.71 (d, *J =* 3.2 Hz, 1H), 6.91 (dd, *J =* 5.1, 3.5 Hz, 1H), 7.17 (dd, *J =* 5.1, 1.0 Hz, 1H), 7.47 (s, 1H), 8.23 (s, 1H) ppm. ^13^C-NMR (CDCl_3_): *δ* 30.7, 34.8, 51.9, 124.5, 126.2, 127.2, 138.4, 157.7 ppm. LC-MS: ELSD pur. 99%, UV pur. 100%; R_t_ = 7.42 min; *m/z*: 305 ([M+H]^+^).

*1-(3,6-Anhydro-1-deoxy-4,5-O-isopropylidene-D-glucitol-1-yl)-4-trifluoroacetamidomethyl-1,2,3-triazole* (**a7b8**). Prepared from 76 mg (0.50 mmol) of **a7** and 126 mg (0.55 mmol) of **b8**. The product was obtained as a pale yellow oil (170 mg, 89%). C_14_H_19_F_3_N_4_O_5_, M = 380.33 g.mol^−1^. FTIR: *ν* 3269, 2994, 1721, 1555,1438, 1197,1185, 1159, 1102, 1056 cm^−1^. ^1^H-NMR (CDCl_3_): *δ* 1.33 (s, 3H), 1.50 (s, 3H), 3.19 (dd, *J =* 5.8, 3.0 Hz, 1H), 3.47 (dd, *J =* 11.0, 2.8 Hz, 1H), 3.64 (s, 1H), 4.07 (d, *J =* 10.7 Hz, 1H), 4.39 (dt, *J =* 6.0, 3.69 Hz, 1H), 4.61 (d, *J =* 7.6 Hz, 2H), 4.74–4.65 (m, 1H), 4.86–4.78 (m, 2H), 7.81 (s, 1H), 7.90 (s, 1H) ppm. ^13^C-NMR (CDCl_3_): *δ* 24.3, 25.8, 29.7, 35.0, 52.8, 69.1, 72.2, 79.6, 80.3, 81.3, 84.4, 112.7, 124.6, 142.4, 157.1 ppm. LC-MS: ELSD pur. 99%, UV pur. 100%; R_t_ = 2.76min; *m/z*: 381 ([M+H]^+^).

*1-Benzyl-4-(tert-butoxycarbonylamino)methyl-1,2,3-triazole* (**a8b1**). Prepared from 78 mg (0.50 mmol) of **a8** and 73 mg (0.55 mmol) of **b1**. The product was obtained as a white solid (130 mg, 90%). C_15_H_20_N_4_O_2_, M = 288.35 g.mol^−1^. m.p. 100–102 ºC. FTIR: *ν* 3236, 3144, 3032, 2974, 1692, 1542, 1289, 1168, 1056 cm^−1^. ^1^H-NMR (CDCl_3_): *δ* 1.40 (s, 9H), 4.38 (d, *J* = 4.6 Hz, 2H), 5.21 (s, 1H), 5.49 (s,2H), 7.23–7.36 (m, 5H), 7.47 (s, 1H) ppm. ^13^C-NMR (CDCl_3_): *δ* 28.3, 36.2, 54.2, 79.6, 128.1, 128.7, 129.1, 134.6, 155.8 ppm. LC-MS: ELSD pur. 98%, UV pur. 100%; R_t_ = 8.17 min; *m/z*: 289 ([M+H]^+^

*4-(tert-Butoxycarbonylamino)methyl-1-ethoxycarbonylmethyl-1,2,3-triazole* (**a8b2**). Prepared from 78 mg (0.50 mmol) of **a8** and 71 mg (0.55 mmol) of **b2**. The product was obtained as a pale green solid (130 mg, 91%). C_12_H_20_N_4_O_4_, M = 284.32 g.mol^−1^. m.p.108–110 ºC. FTIR: *ν* 3394, 3132, 3073, 2970, 1757, 1687, 1517, 1210, 1177, 1056 cm^−1^. ^1^H-NMR (CDCl_3_): *δ* 1.30 (t, *J =* 7.1 Hz, 3H), 1.44 (s, 9H), 4.26 (q, *J =* 7.1 Hz, 2H), 4.42 (d, *J =* 5.8 Hz, 2H), 5.14 (s, 2H), 5.27 (s, 1H), 7.67 (s, 1H) ppm. ^13^C-NMR (CDCl_3_): *δ* 14.1, 28.4, 36.1, 50.9, 62.4, 79.7, 155.9, 166.2 ppm. LC-MS: ELSD pur. 94%, UV pur. 100%; R_t_ = 5.33 min; *m/z*: 285 ([M+H]^+^).

*4-(tert-Butoxycarbonylamino)methyl-1-(3-hydroxypropyl)-1,2,3-triazole* (**a8b3**). Prepared from 78 mg (0.50 mmol) of **a8** and 56 mg (0.55 mmol) of **b3**. The product was obtained as a pale green oil (127 mg, 99%). C_11_H_20_N_4_O_3_, M = 256.31 g.mol^−1^. FTIR: *ν* 3336, 3136, 2965, 2933, 2875, 1696, 1525, 1168, 1048 cm^−1^. ^1^H-NMR (CDCl_3_): *δ* 1.42 (s, 9H), 2.11 (q, *J* = 5.1 Hz, 2H), 3.62 (m,2H), 3.72 (s,1H), 4.50 (t, *J* = 6.6 Hz, 2H), 5.1 (s, 1H), 7.67 (s, 1H) ppm. ^13^C-NMR (CDCl_3_): *δ* 28.4, 32.7, 36.0, 47.1, 58.1, 79.7, 156.1 ppm. LC-MS: ELSD pur. 93%, UV pur. 100%; R_t_ = 2.38 min; *m/z*: 257 ([M+H]^+^).

*4-(tert-Butoxycarbonylamino)methyl-1-(3-trifluoroacetamidopropyl)-1,2,3-triazole* (**a8b4**). Prepared from 78 mg (0.50 mmol) of **a8** and 108 mg (0.55 mmol) of **b4**. The product was obtained as a off-white solid (175 mg, 99%). C_13_H_20_F_3_N_5_O_3_, M = 351.33 g.mol^−1^. m.p.132–134 ºC. FTIR: *ν* 3323, 3127, 3099, 2974, 2933, 2883, 1728, 1704, 1674, 1530, 1210, 1181, 1168, 1048 cm^−1^. ^1^H-NMR (CDCl_3_): *δ* 1.43 (s, 9H), 2.23 (p, *J =* 6.6 Hz, 2H), 3.41 (q, *J =* 6.3 Hz, 2H), 4.64–4.18 (m, 4H), 5.29 (s, 1H), 7.62 (s, 1H), 7.72 (s, 1H) ppm. ^13^C-NMR (CDCl_3_): *δ* 28.4, 29.2, 36.0, 37.1, 47.7, 79.9, 156.0 ppm. LC-MS: ELSD pur. 99%, UV pur. 100%; R_t_ = 5.73 min; *m/z*: 352 ([M+H]^+^).

*4-(tert-Butoxycarbonylamino)methyl-1-(2,2-dimethyl-1,3-dioxolan-4-yl)methyl-1,2,3-triazole* (**a8b5**). Prepared from 78 mg (0.50 mmol) of **a8** and 86 mg (0.55 mmol) of **b5**. The product was obtained as a pale green solid (150 mg, 96%). C_14_H_24_N_4_O_4_, M = 312.37 g.mol^−1^. m.p. 100–102 ºC. FTIR: *ν* 3394, 2983, 2899, 1687, 1512, 1384, 1368, 1272, 1251, 1210, 1168, 1143, 1069 cm^−1^. ^1^H-NMR (CDCl_3_): *δ* 1.34 (s, 3H), 1.38 (s, 3H), 1.44 (s, 9H), 3.74 (dd, *J =* 8.7, 5.5 Hz, 1H), 4.12 (dd, *J =* 8.7, 6.1 Hz, 1H), 4.65–4.28 (m, 5H), 5.25 (s, 1H), 7.70 (s, 1H) ppm. ^13^C-NMR (CDCl_3_): *δ* 25.2, 26.7, 28.4, 36.1, 52.4, 66.4, 74.0, 79.6, 110.2, 155.8 ppm. LC-MS: ELSD pur. 99%, UV pur. 100%; R_t_ = 5.22 min; *m/z*: 313 ([M+H]^+^).

*4-(tert-Butoxycarbonylamino)methyl-1-[(cyclohex-3-en-1-yl)methyl]-1,2,3-triazole* (**a8b6**). Prepared from 78 mg (0.50 mmol) of **a8** and 75 mg (0.55 mmol) of **b6**. The product was obtained as a beige solid (145 mg, 99%). C_15_H_24_N_4_O_2_, M = 292.38 g.mol^−1^. m.p. 86–88 ºC. FTIR: *ν* 3385, 3127, 3019, 2965, 2920, 1696, 1512, 1363, 1272, 1168, 1052 cm^−1^. ^1^H-NMR (CDCl_3_): *δ* 1.44 (s, 9H), 1.88–1.61 (m, 2H), 2.41–1.88 (m, 5H), 4.26 (d, *J =* 7.3 Hz, 2H), 4.40 (d, *J =* 5.5 Hz, 2H), 5.24 (s, 1H), 5.81–5.52 (m, 2H), 7.53 (s, 1H) ppm. ^13^C-NMR (CDCl_3_): *δ* 24.1, 25.2, 28.4, 28.9, 34.7, 36.1, 55.4, 79.6, 124.8, 127.1, 155.9 ppm. LC-MS: ELSD pur. 99%, UV pur. 100%; R_t_ = 8.55 min; *m/z*: 293 ([M+H]^+^).

*4-(tert-Butoxycarbonylamino)methyl-1-[2-(2-thienyl)ethyl]-1,2,3-triazole* (**a8b7**). Prepared from 78 mg (0.50 mmol) of **a8** and 75 mg (0.55 mmol) of **b7**. The product was obtained as a beige solid (150 mg, 97%). C_14_H_20_N_4_O_2_S, M = 308.41 g.mol^−1^. m.p. 92–94 ºC. FTIR: *ν* 3311, 3120, 3095, 3070, 2978, 2933, 2875, 1679,1534, 1447, 1363, 1251, 1164, 1056 cm^−1^. ^1^H-NMR (CDCl_3_): *δ* 1.43 (s, 9H), 3.42 (t, *J =* 7.0 Hz, 2H), 4.36 (d, *J =* 5.9 Hz, 2H), 4.59 (t, *J =* 7.1 Hz, 2H), 5.21 (s, 1H), 6.73 (dd, *J =* 3.4, 0.9 Hz, 1H), 6.91 (dd, *J =* 5.1, 3.5 Hz, 1H), 7.17 (dd, *J =* 5.1, 1.1 Hz, 1H), 7.38 (s, 1H) ppm. ^13^C-NMR (CDCl_3_): *δ* 28.4, 30.8, 36.0, 52.7, 79.6, 124.6, 126.1, 127.0, 138.8, 155.7 ppm. LC-MS: ELSD pur. 99%, UV pur. 100%; R_t_ = 8.20min; *m/z*: 309 ([M+H]^+^).

*1-(3,6-Anhydro-1-deoxy-4,5-O-isopropylidene-D-glucitol-1-yl)-4-(tert-butoxycarbonylamino)methyl-1,2,3-triazole* (**a8b8**). Prepared from 78 mg (0.50 mmol) of **a8** and 75 mg (0.55 mmol) of **b8**. The product was obtained as a beige solid (192 mg, 99%). C_17_H_28_N_4_O_6_, M = 384.44 g.mol^−1^. m.p. 130–132 ºC. FTIR: *ν* 3550, 3406, 2937, 2854, 1683, 1511, 1455, 1375, 1272, 1159, 1106, 1052 cm^−1^. ^1^H-NMR (CDCl_3_): *δ* 1.33 (s, 3H), 1.44 (s, 9H), 1.50 (s, 3H), 3.15 (dd, *J =* 5.9, 3.5 Hz, 1H), 3.57–3.40 (m, 2H), 4.07 (d, *J =* 10.7 Hz, 1H), 4.40 (d, *J =* 5.7 Hz, 2H), 4.56 (dd, *J =* 14.2, 3.5 Hz, 1H), 4.67 (dd, *J =* 14.4, 6.0 Hz, 1H), 4.88–4.76 (m, 2H), 5.25 (s, 1H), 7.72 (s, 1H) ppm. ^13^C-NMR (CDCl_3_): *δ* 24.3, 25.9, 28.4, 36.1, 52.5, 69.2, 72.6, 79.6, 80.3, 81.3, 81.6, 84.4, 112.6, 123.8, 155.8 ppm. LC-MS: ELSD pur. 99%, UV pur. 100%; R_t_ = 3.43 min; *m/z*: 385 ([M+H]^+^).

*Tris [(1-benzyl-1,2,3-triazol-4-yl)methyl]amine* (**a9b1**). Prepared from 69 mg (0.50 mmol) of **a9** and 220 mg (1.65 mmol) of **b1**. The product was obtained as a mixture of mono, bis (11%) and tris (81%) derivatives. C_30_H_30_N_10_, M = 530.64 g.mol^−1^. LC-MS: ELSD pur. 81%, UV pur. 81%; R_t_ = 5.5 min; *m/z*: 531 ([M+H]^+^).

*Tris [(1-ethoxycarbonylmethyl-1,2,3-triazol-4-yl)methyl]amine* (**a9b2**). Prepared from 69 mg (0.50 mmol) of **a9** and 213 mg (1.65 mmol) of **b2**. The product was obtained as a mixture of mono, bis (44%) and tris (47%) derivatives. C_21_H_30_N_10_O_6_, M = 518.53 g.mol^−1^. LC-MS: ELSD pur. 47%, UV pur. 50%; R_t_ = 1.86 min; *m/z*: 519 ([M+H]^+^).

*Tris {[1-(3-hydroxypropyl)-1,2,3-triazol-4-yl]methyl}amine* (**a9b3**). Prepared from 69 mg (0.50 mmol) of **a9** and 167 mg (1.65 mmol) of **b3**. The product was obtained as a mixture of mono, bis (99%) and tris (1%) derivatives. C_18_H_30_N_10_O_3_, M = 434.50 g.mol^−1^. LC-MS: ELSD pur. 0.8%, UV pur. 0.8%; R_t_ = 380 min; *m/z*: 435 ([M+H]^+^).

*Tris {[1-(3-trifluoroacetamidopropyl)-1,2,3-triazol-4-yl]methyl}amine* (**a9b4**). Prepared from 78 mg (0.50 mmol) of **a9** and 108 mg (1.65 mmol) of **b4**. The product was obtained as a mixture of mono, bis (7%) and tris (92%) derivatives. C_21_H_30_F_9_N_13_O_3_, M = 719.58 g.mol^−1^. LC-MS: ELSD pur. 92%, UV pur. 90%; R_t_ = 1.86 min; *m/z*: 720 ([M+H]^+^).

*Tris {[1-(2,2-dimethyl-1,3-dioxolan-4-yl)methyl-1,2,3-triazol-4-yl]methyl}amine* (**a9b5**). Prepared from 78 mg (0.50 mmol) of **a9** and 86 mg (1.65 mmol) of **b5**. The product was obtained as a mixture of mono, bis (11%) and tris (88%) derivatives. C_27_H_42_N_10_O_6_, M = 602.70 g.mol^−1^. LC-MS: ELSD pur. 88%, UV pur. 99%; R_t_ = 1.17 min; *m/z*: 603 ([M+H]^+^).

*Tris ({1-[(cyclohex-3-en-1-yl)methyl]-1,2,3-triazol-4-yl}methyl)amine* (**a9b6**). Prepared from 69 mg (0.50 mmol) of **a9** and 226 mg (1.65 mmol) of **b6**. The product was obtained as a mixture of mono, bis (34%) and tris (61%) derivatives. C_30_H_42_N_10_, M = 542.72 g.mol^−1^. LC-MS: ELSD pur. 61%, UV pur. 62%; R_t_ = 7.82 min; *m/z*: 543 ([M+H]^+^).

*Tris ({1-[2-(2-thienyl)ethyl]-1,2,3-triazol-4-yl}methyl)amine* (**a9b7**). Prepared from 69 mg (0.50 mmol) of **a9** and 225 mg (1.65 mmol) of **b7**. The product was obtained as a mixture of mono, bis (51%) and tris (47%) derivatives. C_24_H_24_N_10_S_3_, M = 590.80 g.mol^−1^. LC-MS: ELSD pur. 47%, UV pur. 50%; R_t_ = 8.05min; *m/z*: 592 ([M+H]^+^).

*Tris {[1-(3,6-anhydro-1-deoxy-4,5-O-isopropylidene-D-glucitol-1-yl)-1,2,3-triazol-4-yl]methyl}amine* (**a9b8**). Prepared from 76 mg (0.50 mmol) of **a9** and 378 mg (1.65 mmol) of **b8**. The product was obtained as a mixture of mono, bis (44%) and tris (53%) derivatives. C_36_H_54_N_10_O_12_, M = 818.89 g.mol^−1^. LC-MS: ELSD pur. 53%, UV pur. 50%; R_t_ = 1.85min; *m/z*: 819 ([M+H]^+^).

*1-Benzyl-4-phenyl-1,2,3-triazole* (**a10b1**). Prepared from 55 mg (0.50 mmol) of **a10** and 73 mg (0.55 mmol)of **b1**. The product was obtained as a beige solid (95 mg, 81%). C_15_H_13_N_3_, M = 235.29 g.mol^−1^. m.p. 128 ºC. FTIR: *ν* 3144, 3037, 2975, 1496, 1451, 1044 cm^−1^. ^1^H-NMR (CDCl_3_): *δ* 3.90 (s, 3H), 5.55 (s, 2H), 7.27–7.36 (m, 5H), 7.99 (s, 1H) ppm. ^13^C-NMR (CDCl_3_): *δ* 52.2, 54.5, 127.3, 128.3, 129.2, 129.3, 133.6, 140.3, 161.1 ppm. LC-MS: ELSD pur. 93%, UV pur. 100%, R_t_ = 8.86 min, *m/z*: 236 ([M+H]^+^, 100%).

*1-Ethoxycarbonylmethyl-4-phenyl-1,2,3-triazole* (**a10b2**). Prepared from 55 mg (0.50 mmol) of **a10** and 71 mg (0.55 mmol) of **b2**. The product was obtained as a white solid (92 mg, 80%). C_12_H_13_N_3_O_2_, M = 231.26 g.mol^−1^. m.p. 104 ºC. FTIR: *ν* 3302, 3140, 3079, 3004, 2950, 1758, 1464, 1082, 1044 cm^−1^. ^1^H-NMR (CDCl_3_): *δ* 1.30 (t, *J* = 7.2 Hz, 3H), 3.95 (s, 3H), 4.28 (q, *J* = 7.2 Hz, 2H), 5.25 (s, 2H), 8.30 (s, 1H) ppm. ^13^C-NMR (CDCl_3_): *δ* 14.4, 51.4, 52.7, 63.2, 129.4, 161.4, 166.0 ppm. LC-MS: ELSD pur. 91%, UV pur. 100%, R_t_ = 8.10 min, *m/z*: 232 ([M+H]^+^, 100%).

*1-(3-Hydroxypropyl)-4-phenyl-1,2,3-triazole* (**a10b3**). Prepared from 55 mg (0.50 mmol) of **a10** and 56 mg (0.55 mmol) of **b3**. The product was obtained as a white solid (88 mg, 87%). C_11_H_13_N_3_O, M = 203.25 g.mol^−1^. m.p. 90.5 ºC. FTIR: *ν* 3315, 3120, 2950, 2875, 1086 cm^−1^. ^1^H-NMR (CDCl_3_): *δ* 2.18 (q, *J* = 6.0 Hz, 2H), 3.67 (t, *J* = 6.0 Hz, 2H), 3.93 (s, 3H), 4.62 (t, *J* = 6.0 Hz, 2H), 8.22 (s, 1H) ppm. ^13^C-NMR (CDCl_3_): *δ* 32.4, 47.5, 52.2, 58.3, 128.2, 139.7, 161.2 ppm. LC-MS: ELSD pur. 98%, UV pur. 100%, R_t_ = 2.95 min, *m/z*: 204 ([M+H]^+^, 100%).

*1-(3-Trifluoroacetamidopropyl)-4-phenyl-1,2,3-triazole* (**a10b4**). Prepared from 55 mg (0.50 mmol) of **a10** and 108 mg (0.55 mmol) of **b4**. The product was obtained as a white solid (132 mg, 88%). C_13_H_13_F_3_N_4_O, M = 298.27 g.mol^−1^. m.p. 159 ºC. FTIR: *ν* 3211, 3045, 2953, 2896, 1721, 1193, 1144 cm^−1^. ^1^H-NMR (CDCl_3_): *δ* 2.30 (t, *J* = 7.2 Hz, 2H), 3.27 (q, *J* = 6.9 Hz, 2H), 4.44 (t, *J* = 7.0 Hz, 2H), 7,33–7,85 (m, 5H), 8.57 (s, 1H) ppm. ^13^C-NMR (CDCl_3_): *δ* 28.7, 36.6, 47.2, 117.7, 121.4, 125.1, 127.8, 128.8, 130.7, 146.2, 156.1 ppm. LC-MS: ELSD pur. 95%, UV pur. 100%, R_t_ = 7.88 min, *m/z*: 299 ([M+H]^+^, 100%).

*1-(2,2-Dimethyl-1,3-dioxolan-4-yl)methyl-4-phenyl-1,2,3-triazole* (**a10b5**). Prepared from 55 mg (0.50 mmol) of **a10** and 86 mg (0.55 mmol) of **b5**. The product was obtained as a beige solid (96 mg, 74%). C_14_H_17_N_3_O_2_, M = 259.31 g.mol^−1^. m.p. 110 ºC. FTIR: *ν* 3144, 2991, 2925, 1260, 1069, 1048 cm^−1^. ^1^H-NMR (CDCl_3_): *δ* 1.32 (s, 3H), 1.37 (s, 3H), 3.73 (dd, *J* = 5.3, 8.7 Hz, 1H), 4.10 (dd, *J* = 6.1, 8.7 Hz, 1H), 4.36–4.56 (m, 3H), 4.74 (s, 2H), 7.73 (s, 1H) ppm. ^13^C-NMR (CDCl_3_): *δ* 25.2, 26.7, 52.3, 66.4, 110.2, 120.9, 125.7, 128.2, 128.8, 130.6, 147.8 ppm. LC-MS: ELSD pur. 97%, UV pur. 100%, R_t_ = 7.96 min, *m/z*: 260 ([M+H]^+^, 100%).

*1-[(Cyclohex-3-en-1-yl)methyl]-4-phenyl-1,2,3-triazole* (**a10b6**). Prepared from 55 mg (0.50 mmol) of **a10** and 75 mg (0.55 mmol) of **b6**. The product was obtained as a brown solid (98 mg, 82%). C_15_H_17_N_3_, M = 239.32 g.mol^−1^. m.p. 93 ºC. FTIR: *ν* 3140, 3074, 3020, 2921, 2842, 1222, 1044 cm^−1^. ^1^H-NMR (CDCl_3_): *δ* 1.32 (m, 2H), 1.75 (m, 2H), 2.02 (m, 2H), 2.20 (m, 1H), 4.27 (d, *J* = 7.3 Hz, 2H), 4.78 (s, 2H), 5.60–5.72 (m, 2H), 7.58 (s, 1H) ppm. ^13^C-NMR (CDCl_3_): *δ* 24.6, 26.3, 29.3, 35.2, 55.8, 120.3, 125.2, 126.1, 127.5, 128.5, 129.2, 131.1, 148.0 ppm. LC-MS: ELSD pur. 94%, UV pur. 100%, R_t_ = 9.37 min, *m/z*: 240 ([M+H]^+^, 100%).

*4-Phenyl-1-[2-(2-thienyl)ethyl]-1,2,3-triazole* (**a10b7**). Prepared from 55 mg (0.50 mmol) of **a10** and 84 mg (0.55 mmol) of **b7**. The product was obtained as a beige solid (96 mg, 76%). C_14_H_13_N_3_S, M = 255.34 g.mol^−1^. m.p. 126 ºC. FTIR: *ν* 3086, 2962, 2924, 1222, 1085 cm^−1^. ^1^H-NMR (CDCl_3_): *δ* 3.42 (t, *J* = 7.1 Hz, 2H), 4.59 (t, *J* = 7.1 Hz, 2H), 4.73 (s, 2H), 6.74 (d, *J* = 3.3 Hz, 1H), 6.91 (dd, *J* = 3.3, 5.0 Hz, 1H), 7.16 (d, *J* = 5.1 Hz, 1H), 7.47 (s, 1H) ppm. ^13^C-NMR (CDCl_3_): *δ* 31.3, 52.2, 120.4, 125.0, 126.1, 126.7, 128.5, 129.2, 131.0, 139.3, 148.0 ppm. LC-MS: ELSD pur. 100%, UV pur. 100%, R_t_ = 8.86 min, *m/z*: 256 ([M+H]^+^, 100%).

*1-(3,6-Anhydro-1-deoxy-4,5-O-isopropylidene-D-glucitol-1-yl)-4-phenyl-1,2,3-triazole* (**a10b8**). Prepared from 55 mg (0.50 mmol) of **a10** and 126 mg (0.55 mmol) of **b8**. The product was obtained as a orange solid (134 mg, 81%). C_17_H_21_N_3_O_4_, M = 331.37 g.mol^−1^. m.p. 89.5 ºC. FTIR: *ν* 3452, 3137, 2987, 2929, 1381, 1277, 1210, 1081 cm^−1^. ^1^H-NMR (CDCl_3_): *δ* 1.35 (s, 3H), 1.52 (s, 3H), 3.19 (dd, *J* = 3.7, 5.8 Hz, 1H), 3.46 (dd, *J* = 3.5, 10.8 Hz, 1H), 4.08 (d, *J* = 10.8 Hz, 1H), 4.47 (dd, *J* = 5.8, 9.3 Hz, 1H), 4.62 (dd, *J* = 3.5, 14.3 Hz, 1H), 4.76 (dd, *J* = 5.7, 14.4 Hz, 1H), 4.80 (dd, *J* = 3.5, 6.2 Hz, 1H), 4.87 (dd, *J* = 3.5, 6.2 Hz, 1H), 7.32–7.85 (m, 5H), 8.01 (s, 1H) ppm. ^13^C-NMR (CDCl_3_): *δ* 24.7, 26.3, 52.9, 69.6, 73.0, 81.0, 81.8, 84.7, 113.0 122.1, 126.1, 128.5, 129.2, 131.0, 148.0 ppm. LC-MS: ELSD pur. 70%, UV pur. 100%, R_t_ = 5.17 min, *m/z*: 332 ([M+H]^+^, 66%), 354 ([M+Na]^+^, 34%).

*1-Benzyl-4-octyl-1,2,3-triazole* (**a11b1**). Prepared from 69 mg (0.50 mmol) of **a11** and 73 mg (0.55 mmol) of **b1**. The product was obtained as a beige solid (108 mg, 80%). C_17_H_25_N_3_, M = 271.41 g.mol^−1^. m.p. 64–66 ºC. FTIR: *ν* 3102, 2958, 2924, 2854, 1483, 1451, 1256, 1197, 1098, 1052, 844 cm^−1^. ^1^H-NMR (CDCl_3_): *δ* 0.86 (t, *J* = 6 Hz, 3H), 1.24–1.28 (m, 12H), 1.58 (t, *J* = 2 Hz, 2H) 5.29 (s, 2H) 7.24–7.34 (m, 5H), 7.36 (s, 1H) ppm. ^13^C-NMR (CDCl_3_): *δ* 14.7, 21.0, 23.1, 29.8, 29.9, 30.0, 30.1, 32.4, 55.4, 128.5, 129.4, 129.6, 135.9 ppm. LC-MS: ELSD pur. 99%, UV pur. 100%; R_t_ = 10.02 min; *m/z*: 273 ([M+H]^+^).

*1-Ethoxycarbonylmethyl-4-octyl-1,2,3-triazole* (**a11b2**). Prepared from 69 mg (0.50 mmol) of **a11** and 71 mg (0.55 mmol) of **b2**. The product was obtained as a brown oil (115 mg, 86%). C_14_H_25_N_3_O_2_, M = 267.37 g.mol^−1^. FTIR: *ν* 3124, 2983, 2958, 1746, 1256, 1206 cm^−1^. ^1^H-NMR (CDCl_3_): *δ* 0.87 (t, *J* = 6 Hz, 3H), 1.28–1.31 (m, 10H), 1.62–1.64 (m, 3H), 1.70 (m, 5H), 4.2 (q, *J* = 7 Hz, 2H), 4.93 (s, 1H), 7.26 (s, 1H) ppm. ^13^C-NMR (CDCl_3_): *δ* 14.4(8), 14.5, 23.1, 26.0, 29.5, 29.5, 29.6, 32.2, 50.6, 63.9, 167.0 ppm. LC-MS: ELSD pur. 90%, UV pur. 100%; R_t_ = 9.54 min; *m/z*: 268 ([M+H]^+^).

*1-(3-Hydroxypropyl)-4-octyl-1,2,3-triazole* (**a11b3**). Prepared from 69 mg (0.50 mmol) of **a11** and 56 mg (0.55 mmol) of **b3**. The product was obtained as a yellow oil (77 mg, 65%). C_13_H_25_N_3_O, M = 239.36 g.mol^−1^. FTIR: *ν* 3347, 3120, 2953, 2937, 1247, 1197, 1118, 1056, 844 cm^−1^. ^1^H-NMR (CDCl_3_): *δ* 0.86 (t, *J* = 6 Hz, 3H), 1.26–1.29 (m, 10H), 1.67 (q, *J* = 3 Hz, 2H) 1.98 (q, *J* = 3 Hz, 2H), 2.52 (t, *J* = 6 Hz, 2H), 3.23 (t, *J* = 9 Hz, 2H), 3.45 (t, *J* = 9 Hz, 2H), 7.36 (s, 1H) ppm. ^13^C-NMR (CDCl_3_): *δ* 14.5, 18.8, 23.07, 29.6, 29.6(8), 29.7, 29.8, 32.3, 33.1, 43.1, 59.3, 84.5, 131.3 ppm. LC-MS: ELSD pur. 97%, UV pur. 100%; R_t_ = 7.82 min; *m/z*: 240 ([M+H]^+^).

*1-(3-Trifluoroacetamidopropyl)-4-octyl-1,2,3-triazole* (**a11b4**). Prepared from 78 mg (0.50 mmol) of **a11** and 108 mg (0.55 mmol) of **b4**. The product was obtained as a pale green solid (165 mg, 99%). C_14_H_25_F_3_N_4_O, M = 334.39 g.mol^−1^. m.p.104–106 ºC. FTIR: *ν* 3207, 3048, 2924, 2854, 1712, 1571, 1463, 1197, 1139 cm^−1^. ^1^H-NMR (CD_3_CN): *δ* 0.87 (q, *J* = 3 Hz, 3H), 1.27–1.32 (m, 10H), 1.60–1.62 (m, 2H), 2.63 (t, *J* = 3 Hz, 2H), 3.27 (q, *J* = 2 Hz, 2H), 3.34–3.37 (m, 2H), 4.34 (t, *J* = 6 Hz, 2H), 7.55 (s, H), 7.67 (s, H) ppm. ^13^C-NMR (CD_3_CN): *δ* 14.3, 21.7, 23.3, 28.6, 29.8, 29.9, 29.9, 30.0, 30.2, 37.7, 48.1 ppm. LC-MS: ELSD pur. 99%, UV pur. 100%; R_t_ = 9.32 min; *m/z*: 352 ([M+NH_4_]^+^).

*1-(2,2-Dimethyl-1,3-dioxolan-4-yl)methyl-4-octyl-1,2,3-triazole* (**a11b5**). Prepared from 78 mg (0.50 mmol) of **a11** and 86 mg (0.55 mmol) of **b5**. The product was obtained as a pale green solid (146 mg, 99%). C_16_H_27_N_3_O_2_, M = 295.43 g.mol^−1^. m.p. 70–72 ºC. FTIR: *ν* 3122, 3064, 2953, 2920, 1461, 1378, 1267, 1057 cm^−1^. ^1^H-NMR (CDCl_3_): *δ* 0.67 (t, *J* = 6 Hz, 3H), 1.06–1.12 (m, 8H), 1.15 (s, 3H), 1.18 (s, 3H), 1.47–1.49 (m, 2H), 2.54 (t, *J* = 6 Hz, 2H), 3.50–3.55 (m, 2H), 3.88–3.92 (m, 2H), 4.19–4.34 (m, 3H), 7.22 (s, 1H) ppm. ^13^C-NMR (CDCl_3_): *δ* 14.2, 22.8, 23.4, 25.4, 26.8, 29.3, 29.4, 29.5, 29.6, 32.0, 52.9, 66.4, 110.2 ppm. LC-MS: ELSD pur. 99%, UV pur. 100%; R_t_ = 9.06 min; *m/z*: 296 ([M+H]^+^).

*1-[(Cyclohex-3-en-1-yl)methyl]-4-octyl-1,2,3-triazole* (**a11b6**). Prepared from 69 mg (0.50 mmol) of **a11** and 75 mg (0.55 mmol) of **b6**. The product was obtained as a brown oil (136 mg, 99%). C_15_H_27_N_3_O_2_, M = 275.44 g.mol^−1^. FTIR: *ν* 3127, 3019, 2933, 2854, 1463, 1375, 1052 cm^−1^. ^1^H-NMR (CDCl_3_): *δ* 0.69 (t, *J* = 4.5 Hz, 3H), 1.08–1.13 (m, 8H), 1.58 (s, 3H), 2.01 (s, 3H), 2.06–2.66 (m, 7H), 4.16 (d, *J* = 9 Hz, 2H), 5.53–5.58 (m, 2H), 7.19 (s, 1H) ppm. ^13^C-NMR (CDCl_3_): *δ* 14.5, 23.0, 24.6, 26.1, 26.2, 29.3, 29.6, 29.7, 29.9, 32.2, 35.1, 55.7, 125.3, 155.9 ppm. LC-MS: ELSD pur. 99%, UV pur. 100%; R_t_ = 10.41 min; *m/z*: 276 ([M+H]^+^).

*4-Octyl-1-[2-(2-thienyl)ethyl]-1,2,3-triazole* (**a11b7**). Prepared from 69 mg (0.50 mmol) of **a11** and 75 mg (0.55 mmol) of **b7**. The product was obtained as a pale green solid (106 mg, 73%). C_16_H_25_N_3_S, M = 291.46 g.mol^−1^. m.p. 72–74 ºC. FTIR: *ν* 3102, 3057, 2962, 2929, 2854, 1455, 1060 cm^−1^. ^1^H-NMR (CDCl_3_): *δ* 0.65 (t, *J* = 13 Hz, 3H), 1.08–1.13 (m, 8H), 1.42 (s, 3H) 2.49 (t, *J* = 6 Hz, 2H) 3.21 (t, *J* = 7.5 Hz, 2H), 4.37 (t, *J* = 7.5 Hz, 3H), 6. 52 (d, *J* = 3 Hz, 1H), 6.72 (t, *J* = 15 Hz, 1H), 6.88 (s, 1H), 6.98 (d, *J* = 6 Hz, 1H) ppm. ^13^C-NMR (CDCl_3_): *δ* 14.5, 23.1, 26.0, 29.6, 29.6, 29.7, 29.9, 31.3, 52.0, 124.9, 126.5, 127.6, 130.2 ppm. LC-MS: ELSD pur. 99%, UV pur. 100%; R_t_ = 9.64 min; *m/z*: 293 ([M+H]^+^).

*1-(3,6-Anhydro-1-deoxy-4,5-O-isopropylidene-D-glucitol-1-yl)-4-octyl-1,2,3-triazole* (**a11b8**). Prepared from 76 mg (0.50 mmol) of **a11** and 126 mg (0.55 mmol) of **b8**. The product was obtained as a pale green solid (128 mg, 70%). C_19_H_33_N_3_O_4_, M = 367.49g g.mol^−1^. m.p.120–122 ºC. FTIR: *ν* 3269, 2994, 1721, 1555,1438, 1197,1185, 1159, 1102, 1056 cm^−1^. ^1^H-NMR (CDCl_3_): *δ* 0.64 (t, *J* = 13 Hz, 3H), 1.19–1.25 (m, 21H), 1.41–1.43 (m, 5H), 2.63 (t, *J* = 3 Hz, 2H), 3.41 (dd, *J* = 7, 13.5 Hz, 2H), 4.01 (dd, *J* = 3, 6 Hz, 2H), 4.33–4.79 (m, 8H), 7.44 (s, 1H) ppm. ^13^C-NMR (CDCl_3_): *δ* 14.5, 23.1, 24.7, 24.8, 29.6, 29.7, 29.7, 29.8, 32.2, 52.7, 69.7, 73.0, 81.0, 81.7, 82.2, 130.5 ppm. LC-MS: ELSD pur. 99%, UV pur. 100%; R_t_ = 9.10 min; *m/z*: 368 ([M+H]^+^).

*1-Benzyl-4-trimethylsilyl-1,2,3-triazole* (**a12b1**). Prepared from 246 mg (0.50 mmol) of **a12** and 67 mg (0.50 mmol) of **b1**. The product was obtained as a pale green solid (82 mg, 71%). C_12_H_17_N_3_Si, M = 231.38 g.mol^−1^. m.p. 74–76 ºC. FTIR: *ν* 3286, 3115, 3061, 2953, 2920, 1674, 1542, 1438, 1318, 1280, 1168 cm^−1^. ^1^H-NMR (CDCl_3_): *δ* 0.11 (s, 9H), 5.56 (s, 2H), 7.24–7.34 (m, 5 H), 7.26 (s, 1H) ppm. ^13^C-NMR (CDCl_3_): *δ* 0.02, 54.6, 129.2, 129.4, 130.2, 136.1 ppm. LC-MS: ELSD pur. 96%, UV pur. 100%; R_t_ = 11.23 min; *m/z*: 232 ([M+H]^+^).

*1-Ethoxycarbonylmethyl-4-trimethylsilyl-1,2,3-triazole* (**a12b2**). Prepared from 246 mg (2.50 mmol) of **a12** and 62 mg (0.50 mmol) of **b2**. The product was obtained as a brown oil (107 mg, 86%). C_9_H_17_N_3_O_2_Si, M = 227.34 g.mol^−1^. FTIR: *ν* 2928, 2854, 1746, 1455, 1372, 1213, 1023 cm^−1^. ^1^H-NMR (CDCl_3_): *δ* 0.12 (s, 9H), 1.12 (t, *J* = 8 Hz, 3H), 4.08 (q, *J* = 1 Hz, 2H), 5.01 (s, 1H), 7.49 (s, 1H) ppm. ^13^C-NMR (CDCl_3_): *δ* 0.02, 15.2, 51.4, 63.5, 131.5, 167.7 ppm. LC-MS: ELSD pur. 99%, UV pur. 100%; R_t_ = 8.50 min; *m/z*: 228 ([M+H]^+^).

*1-(3-Hydroxypropyl)-4-trimethylsilyl-1,2,3-triazole* (**a12b3**). Prepared from 246 mg (0.50 mmol) of **a12** and 51 mg (0.50 mmol) of **b3**. The product was obtained as a pale green solid (80 mg, 73%). C_8_H_17_N_3_OSi, M = 199.33 g.mol^−1^. m.p. 58–60 ºC. FTIR: *ν* 3290, 2920, 2850, 1650, 1461, 1213, 1172, 1049 cm^−1^. ^1^H-NMR (CDCl_3_): *δ* 0.12 (s, 9H), 1.94 (q, *J* = 2 Hz, 2H), 3.44–3.47 (m, 2H), 3.56 (t, *J* = 4.5 Hz, 1H), 4.36 (t, *J* = 6 Hz, 2H), 7.41 (s, 1H) ppm. ^13^C-NMR (CDCl_3_): *δ* 0.02, 34.1, 47.6, 59.7, 130.7 ppm. LC-MS: ELSD pur. 90%, UV pur. 100%; R_t_ = 2.74 min; *m/z*: 200 ([M+H]^+^).

*1-(3-Trifluoroacetamidopropyl)-4-trimethylsilyl-1,2,3-triazole* (**a12b4**). Prepared from 246 mg (2.50 mmol) of **a12** and 79 mg (0.50 mmol) of **b4**. The product was obtained as a pale green solid (160 mg, 99%). C_10_H_17_F_3_N_4_OSi, M = 294.35 g.mol^−1^. m.p. 120–122 ºC. FTIR: *ν* 3186, 3124, 3073, 2962, 1721, 1571, 1185, 1156 cm^−1^. ^1^H-NMR (CD_3_CN): *δ* 0.15 (s, 9H), 1.81 (t, *J* = 3 Hz, 2H), 3.15–3.17 (m, 2H), 4.28 (q, *J* = 3 Hz, 2H), 7.49–7.50 (m, 1H), 7.68 (s, 1H) ppm. ^13^C-NMR (CD_3_CN): *δ* 0.02, 28.7, 36.5, 47.7, 132.6, 157.7 ppm. LC-MS: ELSD pur. 99%, UV pur. 100%; R_t_ = 10.15 min; *m/z*: 335 ([M+MeCN]^+^).

*1-(2,2-Dimethyl-1,3-dioxolan-4-yl)methyl-4-trimethylsilyl-1,2,3-triazole* (**a12b5**). Prepared from 246 mg (2.50 mmol) of **a12** and 79 mg (0.50 mmol) of **b5**. The product was obtained as a yellow oil (92 mg, 66%). C_10_H_19_N_3_O_2_Si, M = 255.39 g.mol^−1^. FTIR: *ν* 2987, 2962, 2895, 1488, 1375, 1247, 1069, 840 cm^−1^. ^1^H-NMR (CDCl_3_): *δ* 0.32 (s, 9H), 1.16 (s, 3H), 1.18 (s, 3H), 3.12–3.20 (m, 2H), 3.57–3.61 (m, 1H), 3.85–3.88 (m, 2H), 4.28–4.31 (m, 3H), 7.63 (s, 1H) ppm. ^13^C-NMR (CDCl_3_): *δ* 0.02, 26.4, 27.4, 54.0, 128.2, 128.2 ppm. LC-MS: ELSD pur. 92 %, UV pur. 100%; R_t_ = 9.50 min; *m/z*: 296 ([M+MeCN]^+^).

*1-[(Cyclohex-3-en-1-yl)methyl]-4-trimethylsilyl-1,2,3-triazole* (**a12b6**). Prepared from 246 mg (2.50 mmol) of **a12** and 52 mg (0.50 mmol) of **b6**. The product was obtained as a brown oil (128 mg, 99%). C_12_H_21_N_3_Si, M = 235.41 g.mol^−1^. FTIR: *ν* 2924, 2850, 1455, 1251, 840 cm^−1^. ^1^H-NMR (CDCl_3_): *δ* 0.32 (s, 9H), 1.30–1.33 (m, 2H), 1.99–2.00 (m, 2H), 2.06–2.09 (m,2H), 4.29 (d, *J* = 3 Hz, 2H), 5.63–5.65 (m, 1H), 5.67–5.69 (m, 1H), 7.49 (s, 1H) ppm. ^13^C-NMR (CDCl_3_): *δ* 0.02, 25.3, 27.0, 35.9, 55.7, 128.0, 128.2 ppm. LC-MS: ELSD pur. 90%, UV pur. 100%; R_t_ = 10.15 min; *m/z*: 276 ([M+MeCN]^+^).

*4-Trimethylsilyl-1-[2-(2-thienyl)ethyl]-1,2,3-triazole* (**a12b7**). Prepared from 246 mg (2.50 mmol) of **a12** and 77 mg (0.50 mmol) of **b7**. The product was obtained as a beige solid (87 mg, 63%). C_11_H_17_N_3_SSi, M = 251.43 g.mol^−1^. m.p. 76–78 ºC. FTIR: *ν* 3095, 2962, 2904, 2857, 1251, 1197, 840 cm^−1^. ^1^H-NMR (CDCl_3_): *δ* 0.28 (s, 9H), 3.42 (t, *J* = 6 Hz, 2H), 4.63 (t, *J* = 6 Hz, 2H), 6.71 (d, *J* = 3 Hz, 1H), 6.91 (t, *J* = 6 Hz, 1H), 7.18 (d, *J* = 6 Hz, 1H), 7.32 (s, 1H) ppm. ^13^C-NMR (CDCl_3_): *δ* 0.02, 32.2, 52.3, 125.6, 127.3, 128.7, 130.4, 140.1 ppm. LC-MS: ELSD pur. 95%, UV pur.100%; R_t_ = 9.02 min; *m/z*: 292 ([M+MeCN]^+^).

*1-(3,6-Anhydro-1-deoxy-4,5-O-isopropylidene-D-glucitol-1-yl)-4-trimethylsilyl-1,2,3-triazole* (**a12b8**). Prepared from 246 mg (2.50 mmol) of **a12** and 115 mg (0.55 mmol) of **b8**. The product was obtained as a yellow oil (118 mg, 66%). C_14_H_25_N_3_O_4_Si, M = 327.46 g.mol^−1^. FTIR: *ν* 3451, 2924, 2857, 1375, 1272, 1210, 1098, 804 cm^−1^. ^1^H-NMR (CDCl_3_): *δ* 0.21 (s, 9H), 1.12 (s, 1H), 1.25 (s, 1H), 2.95 (m, 1H), 3.25–3.31 (m, 3H), 3.86–3.89 (m, 3H), 3.99 (t, *J* = 6 Hz, 1H), 4.41 (m, 4H), 4.66–4.68 (m, 2H), 7.54 (s, 1H) ppm. ^13^C-NMR (CDCl_3_): *δ* 0.02, 25.4, 25.5, 53.0, 70.3, 73.7, 81.7, 82.4, 83.0, 113.6 ppm. LC-MS: ELSD pur. 99%, UV pur. 100%; R_t_ = 4.94 min; *m/z*: 328 ([M+H]^+^).

## 4. Conclusions

We have presented in this article our findings concerning a polymer-supported copper (I) catalyst for the Huisgen reaction. The results include the study of the polymeric catalyst in terms of loading, efficiency of different samples, its use in different solvents and catalytic levels. The catalyst was found to be more efficient in apolar aprotic solvents at 6–8 mol%. The catalyst can be recycled at least five time without loss of activity, and seems to be stable enough to use it straight from the shelf over a long time. Kinetics of this heterogeneous catalyst was compared to a homogeneous version of it. It was found that the solid-phase catalyst was two times slower than the soluble one, the reactions being complete in 6 hours, but it was giving better results due to the simple product isolation procedure. The catalyst was finally used in automated synthesis of a 96-triazole library. It gave very good results for most of the awaited compounds having a wide scope, but with some limitations for amine bearing alkynes, as an example. Despite this imitation, Amberlyst A-21•CuI is a very efficient tool for the quick preparation of the triazole nucleus with an isolation procedure by simple filtration and evaporation.

Amberlyst A-21•CuI has already found other applications in triazole synthesis in solvent-free approaches, modular flow reactor chemistry - directly from alkynes or *via* a sequential Seyferth-Gilbert homologation/A-21•CuI catalyzed Huisgen reaction, one-pot synthesis of triazoles with *in situ* preparation of azides and synthesis of triazole-containing macrocycles, as well as formation of α-alkylidene cyclic carbonates from propargyl alcohols in super critical carbon dioxide [46,47,48,49,50,51]. Amberlyst A-21 was also used to support other metals, such as ytterbium and palladium, opening the way to other polymer-supported catalysts based of this low cost commercially available macroreticular resin [52,53,54].

We are sure that this approach and catalysts will find other very interesting applications in several reactions where a metallic catalyst is needed, improving the tools of the organic chemist in both regular synthetic and click chemistry.

## Figures and Tables

**Table 1:**
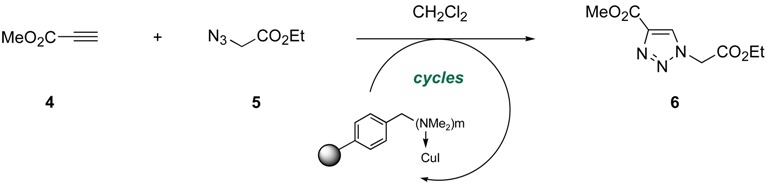
Recycling A-21•CuI in the synthesis of the triazole **6** starting from **4** and **5**. ^a^

Entry	Cycle delay ^b^ (days)	Yield ^c^ %	Entry	Cycle delay ^b^ (weeks)	Yield ^c^ %
1	0	99	7	0	99
2	1	99	8	1	99
3	2	99	9	2	99
4	3	99	10	3	99
5	4	99	11	4	99
6	5	99	12	5	99

^a^ Run in 1 mL methylene chloride stirring at room temperature for 7 hours on 0.5 mmol **4** and 0.55 mmol **5**. ^b^ The catalyst was washed at the end of reaction, dry under vacuum and kept in a vial until its next use. ^c^ Isolated yield of **6**.

**Table 2:**
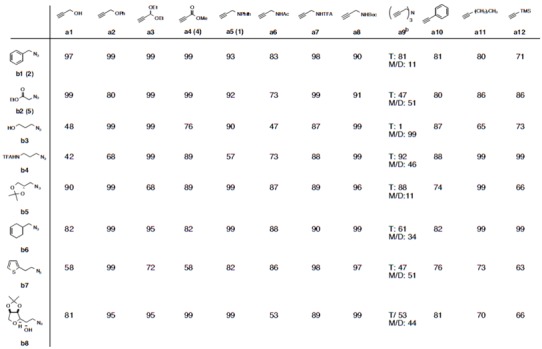
Parallel synthesis of 96 triazoles using Amberlyst A-21-CuI on a ChemSpeed ASW-2000 with 8 azides and 12 Alkynes. ^a^

^a^ See Scheme 4. Isolated or purified yieds. ^b^ Mixtures of tristriazoles (T) and mono-etdi-triazoles (MD) of triporpargylamine
